# Frontal and parietal EEG alpha asymmetry: a large-scale investigation of short-term reliability on distinct EEG systems

**DOI:** 10.1007/s00429-021-02399-1

**Published:** 2021-10-21

**Authors:** Dorothea Metzen, Erhan Genç, Stephan Getzmann, Mauro F. Larra, Edmund Wascher, Sebastian Ocklenburg

**Affiliations:** 1 Department of Biopsychology, Faculty of Psychology, Institute of Cognitive Neuroscience, Ruhr University Bochum, Universitätsstraße 150, 44801 Bochum, Germany; 2 Department of Psychology and Neurosciences, Leibniz Research Centre for Working Environment and Human Factors, Technical University of Dortmund (IfADo), 44139 Dortmund, Germany; 3 Department of Ergonomics, Leibniz Research Centre for Working Environment and Human Factors, Technical University of Dortmund (IfADo), 44139 Dortmund, Germany; 4 Department of Psychology, Medical School Hamburg, 20457 Hamburg, Germany

**Keywords:** Electroencephalogram (EEG), Frontal alpha asymmetry, Parietal alpha asymmetry, Reliability, Handedness

## Abstract

EEG resting-state alpha asymmetry is one of the most widely investigated forms of functional hemispheric asymmetries in both basic and clinical neuroscience. However, studies yield inconsistent results. One crucial prerequisite to obtain reproducible results is the reliability of the index of interest. There is a body of research suggesting a moderate-to-good reliability of EEG resting-state alpha asymmetry, but unfortunately sample sizes in these studies are typically small. This study presents the first large-scale short-term reliability study of frontal and parietal EEG resting-state alpha asymmetry. We used the Dortmund Vital Study data set containing 370 participants. In each participant, EEG resting state was recorded eight times, twice with their eyes opened, twice with their eyes-closed, each on two different EEG systems. We found good reliability of EEG alpha power and alpha asymmetry on both systems for electrode pairs. We also found that alpha power asymmetry reliability is higher in the eyes-closed condition than in the eyes-open condition. The frontomedial electrode pair showed weaker reliability than the frontolateral and parietal electrode pairs. Interestingly, we found no population-level alpha asymmetry in frontal electrodes, one of the most investigated electrode sites in alpha asymmetry research. In conclusion, our results suggest that while EEG alpha asymmetry is an overall reliable measure, frontal alpha asymmetry should be assessed using multiple electrode pairs.

**Supplementary Information:**

The online version contains supplementary material available at 10.1007/s00429-021-02399-1.

## Introduction

For decades, researchers have investigated frontal EEG alpha band (8–13 Hz) asymmetry and its role in psychopathology, motivation, and personality (Allen et al. 2018; Davidson et al. 1979; Gable et al. 2018; Harmon-Jones and Gable 2018; Reznik and Allen 2018). Using the search term “alpha asymmetry” on the scientific search engine PubMed yields almost 2000 hits (February 2021), reflecting the large body of literature that has accumulated on this specific form of hemispheric asymmetries.

Alpha has been proposed to have an inverse relationship with brain activity (Pfurtscheller et al. 1996). Thus, relatively higher right to left alpha power has commonly been interpreted as relatively higher left to right activity, and vice versa (Reznik and Allen 2018). To determine the extent of individual EEG alpha asymmetry, a laterality quotient (LQ) is calculated by subtracting left alpha power from right alpha power (ln[R] − ln[L]) (Reznik and Allen 2018). This way, positive LQ values represent higher relative right alpha power (left activity), while negative LQ values represent higher relative left alpha power (right activity). The amount of EEG alpha activity in the brain is influenced by the eye-status of the participant. In healthy subjects, EEG alpha activity is more pronounced when eyes are closed than when eyes are open (Barry et al. 2007). This decrease in alpha activity is thought to represent an increased activity of the visual system that is activated once eyes are opened and visual information is processed (Barry et al. 2007).

Frontal EEG alpha asymmetry has been associated with various forms of psychopathology, developmental disorders, as well as with interindividual variation in affective style and personality parameters (Reznik and Allen 2018). A vast body of research has associated major depression disorder (MDD) (Allen et al. 2018) with increased left alpha asymmetry during rest (Debener et al. 2000; Metzger et al. 2004; Stewart et al. 2010) as well as during emotional tasks (Stewart et al. 2011a, 2014). These effects can also be found in people with genetic risks for affective disorders (Christou et al. 2016), post-traumatic stress disorder (PTSD) (Meyer et al. 2015, 2018), or remitted MDD (Stewart et al. 2010). Alpha asymmetry may also be linked to responding to antidepressants like serotonin reuptake inhibitors, as responders to the treatment show relative higher left frontal activity, while non-responders show relative higher right frontal activity (Bruder et al. 2008). Alpha asymmetry may additionally be altered in people with attention deficit hyperactivity disorder (ADHD), who show relatively higher left frontal activity than controls (Alperin et al. 2019; Hale et al. 2009).

The reason why frontal alpha asymmetry may be altered in affective and executive disorders could be the frontal lobes’ role in motivational and affective traits in general (Davidson 1998). An especially important part of our personality is how we manage and regulate approach and withdrawal toward or away from stimuli (Gable et al. 2018). Approach-related behavior seems to be linked to higher left frontal activity, while withdrawal-related behavior is linked to higher right frontal activity (Coan and Allen 2003; Grimshaw and Carmel 2014; Harmon-Jones and Allen 1997; Koslov et al. 2011; Sutton and Davidson 1997). Higher left-sided frontal activity has also been linked to positive affect and wellbeing (Papousek et al. 2019; Sutton and Davidson 2000; Urry et al. 2004; Wheeler et al. 1993), better emotion regulation (Jackson et al. 2003; Papousek et al. 2012), better stress regulation (Lewis et al. 2007; Quaedflieg et al. 2015; Zhang et al. 2018), and higher reward responsiveness (Pizzagalli et al. 2005). Lesion studies have indicated that the source of hemispheric alpha asymmetry in depression lies in a deficit in left frontal functioning (Narushima et al. 2003). These results have recently been confirmed by a study using exact low-resolution brain electromagnetic tomography in a large sample: subjects with a history of depression showed reduced left-sided activity in the precentral gyrus and the midfrontal gyrus when compared to subjects without a history of depression (Smith et al. 2018). Thus, decreased left-sided frontal activity in MDD and PTSD may reflect a tendency toward withdrawal-related behavior and negative affect, while increased left-sided frontal activity in ADHD may reflect a tendency toward high approach-related behavior (Meyer et al. 2018; van der Vinne et al. 2017). Furthermore, a recent study found that frontal alpha asymmetries in socially avoidant mothers during emotion-inducing conditions are able to predict their child’s frontal asymmetry pattern (Krzeczkowski et al. 2020). Thus, individual differences in frontal asymmetry patterns may represent a neural mechanism through which withdrawal tendencies are passed onto the next generation. Overall, altered frontal hemispheric asymmetry may be linked to vulnerability for psychopathology and not to any disorders themselves (Ocklenburg et al. 2019).

Additionally, alpha asymmetries have been associated with another important aspect of human brain asymmetry: handedness. A recent meta-analysis with over 2 million subjects showed that 81.9–90.7% of the population is right-handed, depending on the measurement of handedness (Papadatou-Pastou et al. 2020). More interestingly, handedness has been linked to other hemispheric asymmetries. One of the most prominent examples is that left-handers show more right-sided or bilateral language lateralization than right-handers (Knecht et al. 2000; Woodhead et al. 2021). Handedness has also been linked to performance in several tasks: in auditory coordination tasks, participants respond faster to stimuli presented on the side contralateral to their preferred hand (Ocklenburg et al. 2010). Similarly, in arithmetic processing tasks, left-handers show stronger functional right-sided activity than right-handers (Artemenko et al. 2020). Furthermore, approach motivation was found to be associated with left-hemispheric activity in right-handers and right-hemispheric activity in left-handers (Brookshire and Casasanto 2012). Related to approach and withdrawal motivation, left-handers have also shown to be more likely to develop depressive symptoms than right-handers (Denny 2009). Thus, handedness is linked to two of the most researched behavioral correlates of EEG alpha asymmetry. However, research concerning the association of handedness and neurophysiological forms of asymmetry is relatively rare. Early studies examining alpha power asymmetry in the resting state in right-handers found a higher left-sided activation in frontomedial (F3/F4) as well as central (C3/C4) electrode sites in stronger right-handers (Papousek and Schulter 1999). Similarly, a second EEG alpha power asymmetry study reported that non-right-handers show greater frontal right-hemispheric activation than right-handers (Propper et al. 2012). A study by Ocklenburg et al. (2019) used a questionnaire to measure handedness to investigate its relationship to EEG alpha asymmetry at rest. In accordance with previous findings, they found that stronger right-handedness predicted greater rightward alpha asymmetry, or greater left activity. Similarly, a study by Packheiser et al. (2020) used a mobile EEG to measure alpha asymmetry, while their participants performed manual tasks. In accordance with Ocklenburg et al. (2019), they found that right-handed subjects showed higher rightward alpha asymmetry during manual tasks, while left-handed subjects showed higher leftward alpha asymmetry during manual tasks. Thus, EEG alpha asymmetry appears to be correlated to behavioral laterality measures and can distinguish between right- and left-handers. Furthermore, alpha asymmetry seems to be associated with both hand preference as measured by a questionnaire and actual manual performance.

Another—often neglected—neurophysiological measure in alpha asymmetry research is parietal alpha asymmetry (Stewart et al. 2011b). Opposite to frontal activity, depressive symptoms have been attributed to higher relative left-sided activity in parietal areas (Baik et al. 2019; Henriques and Davidson 1990; Stewart et al. 2011b). This may be due to parietal involvement in emotion regulation, as a study has shown that children that show less positive emotion also show less pronounced right-hemispheric parietal activity (Shankman et al. 2005). Thus, it has been proposed that—in addition to frontal asymmetry—parietal asymmetry may play a part in behavioral approach/behavioral inhibition systems, as well (Schiltz et al. 2018).

However, not all results concerning alpha asymmetry effects on psychopathology and personality yield the same results and there are considerably inconsistencies between studies (Bruder et al. 1997; Jesulola et al. 2015; Nusslock et al. 2015; van der Vinne et al. 2017). For instance, a meta-analysis investigating the relationship between frontal alpha asymmetry and MDD found no significant link between the two (van der Vinne et al. 2017), indicating that the relationship between alpha asymmetry and depression is not as reliable as previously thought. Several methodological issues have been suggested as possible reasons for these inconsistencies, like duration of the EEG recordings, operationalization of diagnosis, and symptoms or age (Thibodeau et al. 2006). Furthermore, it has been noticed that alpha asymmetries during emotionally demanding situations or tasks may be a much better indicator for affective disorders than alpha asymmetries during rest (Coan et al. 2006; Stewart et al. 2014) and that some symptom clusters may be better suited for prediction than others (Nusslock et al. 2015).

One essential prerequisite for replicable results is the reliability of EEG alpha asymmetry. For a long time, alpha asymmetries have been regarded as a trait-like quality (Hagemann et al. 2002) that should not change considerably over time. Several studies have investigated test–retest reliability of alpha asymmetry on different sites over different periods of time, yielding moderate-to-good results, comparable to other laterality measures (Voyer 1998). In healthy samples, midfrontal EEG alpha asymmetry reliability 3 weeks apart yielded correlation coefficients between 0.53 and 0.66 (Davidson et al. 1992), frontal alpha asymmetry 1 month apart yielded a correlation coefficient of 0.57 (Winegust et al. 2014), and parietal alpha asymmetry 12 years apart yielded correlation coefficients of 0.57 (Tenke et al. 2018). Retest reliability in depressed samples of frontal alpha 3 months apart yielded a correlation coefficient of 0.61 (Gold et al. 2013). A study investigating a mixed sample found correlation coefficients between 0.54 and 0.60 for frontolateral, frontomedial, and parietal alpha asymmetries and no differences in test–retest reliability between depressed and non-depressed subjects (Vuga et al. 2006). Interestingly, a recent study investigated healthy subjects during an emotional face processing task 1 week apart. They found considerable reliability differences between recording sites (Koller-Schlaud et al. 2020). While frontolateral and parietomedial sites yielded acceptable reliability (*r* = 0.63–0.73), frontomedial and parietolateral sites yielded lower reliability (*r* = 0.30–0.45). Considering these effect sizes, it appears plausible that EEG alpha asymmetry is not entirely a trait-like quality, but may be influenced by state-dependent variables, as well (Hagemann et al. 2002; Peterson and Harmon-Jones 2009).

One important factor to keep in mind when interpreting these studies is that they investigate the reliability of a difference score, which are thought to be less reliable than the scores they are calculated from (Caruso 2004; Cronbach and Furby 1970). This is due to the difference scores containing measurement errors from two measurements (Clayson et al. 2021). It has even been a point of argument if difference scores can be reliable at all (Trafimow 2015). What can be said is that high reliability scores comparable to—for instance—absolute alpha power are not to be expected from studies investigating alpha power asymmetry. Therefore, when keeping this in mind, research suggests an overall good short-term reliability for EEG alpha asymmetry.

In this regard, it should be noted that the study of Koller-Schlaud et al. (2020) had a rather small sample size (*n* = 23). This may also be a critical point of the other mentioned studies investigating reliability of EEG alpha asymmetry, in which sample sizes range from 10 (Winegust et al. 2014) to 99 (Vuga et al. 2006). This may be especially critical considering the present critique concerning small sample sizes and effect sizes in the light of replication crisis, which is an important topic concerning all of neuroscience (Button et al. 2013) and laterality research (Brederoo et al. 2019; Brysbaert 2019; Ocklenburg et al. 2020). Another important point is that none of the above-mentioned studies has systematically investigated the reliability of alpha asymmetries between eyes-open and eyes-closed recordings. The studies investigating resting-state EEG have either used an eyes-open and eyes-closed mixed design, calculating the mean over the whole recording (Davidson et al. 1992; Winegust et al. 2014) or investigated reliability within eyes-closed and eyes-open recordings but not between them (Tenke et al. 2018; Vuga et al. 2006). Thus, the reliability of EEG asymmetry between different eye-status conditions remains uninvestigated.

The aim of the present study was to investigate the short-term reliability of frontal and parietal alpha asymmetries and to test whether previous results can be replicated in a large sample of 370 healthy adults. In each participant, EEG resting state was recorded eight times, four times each on two different EEG systems. On each of the two systems, EEG resting state was recorded two times, while the participant had their eyes-open and two times, while they had their eyes-closed. Compared to previous reliability studies on EEG resting-state asymmetries, our study has a substantially larger sample sizes, enabling a much more robust statement concerning the short-term reliability of EEG asymmetry measures. Considering EEG asymmetry’s immense popularity in clinical as well as basic research, insight into its reliability is crucial.

Taken together, we first aimed to replicate overall leftward frontal alpha asymmetry (van der Vinne et al. 2017; Winegust et al. 2014) and rightward parietal alpha asymmetry in healthy subjects (Ocklenburg et al. 2019). Second, we aimed to replicate alpha asymmetry’s association with handedness as reported by the previous studies (Ocklenburg et al. 2019; Packheiser et al. 2020), with stronger right-handedness predicting greater rightward alpha asymmetry. Third, we wanted to investigate, if reliability of alpha power is different between hemispheres or frontal and parietal brain regions as reported by Koller-Schlaud et al. (2020). Fourth, we wanted to investigate the effect of eye-status on alpha power and on alpha asymmetry and if this effect differs between hemispheres or brain regions. Finally, we wanted to further investigate the effect of eye status on reliability.

## Methods

### Participants

Data were obtained from the Dortmund Vital Study, an ongoing large-sample cohort study on the development of cognitive functions over a wide age range, carried out by the Leibniz Research Centre for Working Environment and Human Factors at the Technical University Dortmund (IfADo). The participants were recruited from local colleges, companies, and public institutions, and through advertisements in newspapers and public media. Data of a total of 583 participants were available. After controlling for outliers, depressive symptoms (see “Statistical analysis”) and excluding all participants with missing EEG data (see “Statistical analysis”), the number of participants analyzed was 370 (220 females, 343 right-handers). They ranged from 20–70 years of age (*M* = 44.46, SD = 14.1). All participants gave their written informed consent before any study protocol was commenced. The study conformed to the Code of Ethics of the World Medical Association (Declaration of Helsinki) and was approved by the local Ethical Committee of IfADo.

### Behavioral measures

The participants hand preference was measured using the Edinburgh Handedness Inventory (EHI) (Oldfield 1971). In this questionnaire, participants are asked to answer ten items regarding several everyday tasks—like writing or throwing—and which hand they are using for this task. The lateralization quotient (LQ) was measured using the following formula LQ [(R − L)/(R + L)] × 100, with R being the sum of right-hand responses and L being the sum of left-hand responses. Hand performance was measured using the Pegboard Test (Francks et al. 2002). In this task, participants sit in front of a board with ten holes on the side of the board nearest and furthest away from the participant. On one side, the holes are filled with little sticks that the participants have to move from this side of the board to the other as fast as possible, using only their right or their left hand. Pegboard lateralization quotient (PegQ) was calculated using the following formula PegQ = [2 × (L − R)/(L + R)], with R being the average time the participant needed to complete the task with their right hand and L being the average time the participant needed to complete the task with their left hand. Participants completed the EHI at home (together with other questionnaires), the Pegboard test was carried out at the first recording day before the EEG testing started.

### Procedure, EEG recording, and pre-processing

The ongoing Dortmund Vital Study includes a series of EEG-based mental tasks in which different cognitive functions are tested. These tasks were performed on 2 different days. The time period between day 1 and day 2 varies and was 57.09 days on average (SD 89; range 1–906) for the cohort analyzed in the present study. The test session on each day lasted about 2 h. Before and after the test session, the EEG resting state was measured, so EEG session 1 and EEG session 2 were always about 2 h apart. In each EEG session, we measured EEG resting state with eyes-closed and with eyes-open, each recording lasting about 2 min each. The eyes-closed condition was always recorded prior to the eyes-open condition.

The recordings from the first day were conducted on a different EEG system than on the second day. This has historical reasons. The tasks on day 1 were arranged in this form specifically for the Dortmund Vital Study and a state-of-the-art amplifier was used. The tasks on day 2 had already been performed by a larger number of participants long before the start of the Dortmund Vital Study, and same amplifier was used on day 2 to achieve better comparability with the previous studies. Thus, EEG on day 1 was recorded using a 64-channel actiCap system (Brain Products GmbH; Munich, Germany) with 1000 Hz sampling rate, while EEG on day 2 recorded using a 32-channel BioSemi system (BioSemi B.V.; Amsterdam, The Netherlands) with a 2048 Hz sampling rate. The two systems mainly differ in the form of the reference used: for the actiCap system, we used FCz as online reference. The BioSemi system uses Common Mode Sense (CMS) active electrode and a Driven Right Leg (DRL) passive electrode. These two electrodes form a feedback loop, which drives the average potential of the subject. Reference and the ground electrodes are integrated in the CLS und DLR loop. Electrodes on both systems were placed according to the international 10–20 system. Impedances were kept below 10 kΩ. EEG signal processing was performed in Matlab 2018b (The MathWorks Inc., Natick, Massachusetts) using functions of the EEGLab toolbox (Delorme and Makeig 2004). The signal was band-pass filtered (1–30 Hz) before corrupted channels were identified and removed based on kurtosis and probability criteria. Subsequently, the data were re-referenced to the average of all electrodes, segmented into 2 s epochs. Corrupted epochs were automatically identified and removed. On average, 94.1% (SD = 4.6) of epochs were retained for EEG system 1 and 96.1% (SD = 4.1) for EEG system 2. Ocular artifacts were removed via independent component analysis. Components representing eye-related activity were identified using ICLabel (Pion-Tonachini et al. 2019) and excluded if classified as eye components with a probability > 80%. Epochs were extracted with 50% overlap using a Hamming window and a Fast Fourier Transform was applied to determine Alpha band (8–13 Hz) power separately for eyes-open and eyes-closed conditions within each session and day. EEG frequency spectra of investigated electrodes can be found in supplementary Fig. 1.

Additionally, we have analyzed the EEG data without controlling for eye movement artifacts. This was done, because the previous research suggested that the effect of eye movement artifacts on EEG alpha power is minor and that controlling for eye movements artifacts in data pre-processing can be omitted (Hagemann and Naumann 2001). However, there is also a line of research that recommends controlling for eye movement artifacts (Smith et al. 2017). In EEG alpha power asymmetry research, there is no consistency in regard to this matter (Coan and Allen 2003; Meyer et al. 2018; Stewart et al. 2014). The results can be found in supplements (supplement 8: results without controlling for ocular artifacts in the data analysis; suppl. Figure 3; suppl. Table 12–13) and are discussed in the discussion of this manuscript.

### Statistical analysis

All calculations were conducted in RStudio (1.4.1103) using R 4.0.3 (2020-10-10). The complete cohort included 583 individuals and the analyzed cohort included 370 subjects (see above). To prepare the data, we first identified outliers of alpha power values. Outliers were indicated by deviating at least three standard deviations from the mean. We had to exclude 22 subjects. Second, we wanted to control for possible influences of depressive symptoms. For this, we used available values of the German version of the Beck’s Depression Inventory II (BDI II) (Beck et al. 1996; Hautzinger et al. 2006). In accordance with the manual (Hautzinger et al. 2006), we excluded all subjects with BDI scores higher than 19, leaving us with subjects that have been classified with either no depressive symptoms or only light depressive symptoms. We had to exclude 23 subjects. Third, we excluded all participants that had any missing values on one of the four asymmetry values investigated in our manuscript. This was done, because repeated-measure ANOVAs do not allow missing values. We had to exclude 168 more subjects. One of the main reasons for the high number of incomplete data sets was that our data set is part of an ongoing longitudinal study. Thus, not all participants with data from the first recording day had already been tested a second time. Our final sample included 370 subjects.

First, we examined the distribution of handedness (see “Distribution of hand preference and hand skill data”) and the distribution of EEG alpha asymmetry (see “Distribution of EEG alpha asymmetry data”). Here, we used Bonferroni-corrected *t*-test against 0 to determine if EEG alpha asymmetry was significantly different from zero. The distribution of seven additional electrode pairs on other scalp sites (Fp1/Fp2, FC3/FC4, CP3/CP4, T7/T8, C3/C4, PO3/PO4, and O1/O2) can be found in the supplements (suppl. Figure 2).

Second, we calculated intra-class correlation (ICC) of all four recording per electrodes and EEG system (see “Reliability of alpha power”), as ICC is a more accurate measurement of reliability than simple correlation coefficients (Koo and Li 2016; Winegust et al. 2014). Following guidelines for choosing the appropriate model of ICC and previous research using ICC for test–retest reliability of EEG data, we used an ICC (3,1) two-way mixed effect model (Koo and Li 2016; van der Velde et al. 2019).

Third, we conducted a 2 × 2 × 2 repeated-measure ANOVA, with the dependent variable being EEG alpha power and the independent variables being eye-status (eyes-open, eyes-closed), system (system 1, system 2), and hemisphere (left, right) (see “Effect of eye-status and hemisphere on EEG alpha power”).

Fourth, we calculated ICC for alpha asymmetry (see “Reliability of EEG resting-state alpha asymmetry”). The EEG alpha AIs (Asymmetry Indices) were determined using the standard formula (ln[right electrode] − ln[left electrode]) (Reznik and Allen 2018). Positive AIs therefore reflect higher relative EEG alpha power in the right hemisphere, and negative AIs reflect higher relative EEG alpha power in the left hemisphere. ICC of additional electrode pairs on other scalp sites can be found in the supplements (suppl. Figure 2, suppl. table 11).

Fifth, we conducted a 4 × 2 × 2 ANOVA to investigate the effect of electrode pair (F3/F4, F7/F8, P3/P4, P7/P8), system (system 1, system 2), and eye-state (eyes-open, eyes-closed) on EEG alpha asymmetry. The majority of alpha power recordings were not normally distributed (Kolmogorov–Smirnov tests, *p* < 0.001). Even though the non-normality of our data violated one of the assumptions of ANOVA, we have decided to carry out our analysis due to our large-sample size and *F* test’s general robustness under departures from normality (Pearson 1931; Schmider et al. 2010). Finally, we correlated EEG alpha asymmetry and handedness. In this analysis, we used Bayesian correlation conducted with the R package *BayesFactor* (Morey and Rouder 2018). This was done due to the low correlation between handedness measures and alpha power asymmetry. This way, we were able to make assumptions about the evidence in favor or against the null hypothesis, instead of just being able to reject the null hypothesis.

There is a large body of research showing that aging has a considerable effect on lateralization of brain activity (Hirnstein et al. 2013; Ocklenburg and Güntürkün 2018). Alpha power and alpha asymmetry have been shown to be affected by aging as well, with older adults showing reduced hemispheric asymmetry than younger adults (Deiber et al. 2013; Hong et al. 2015; Huizeling et al. 2021; Kolev et al. 2002; Vaden et al. 2012). Due to the large age range in our sample (20–70) we have also conducted the analysis concerning reliability separately for young adults (age 20–35, *M* = 27.52, SD = 4.09, *n* = 120), middle-aged adults (age 36–55, *M* = 47.25, SD = 5.71, *n* = 163), and older adults (age 56–70, *M* = 62.6, SD = 4.48, *n* = 87). We also investigated the effect of age on alpha power and alpha power asymmetry. The results of these analysis can be found in the supplements (suppl. Table 5–10).

## Results

### Distribution of hand preference and hand skill data

**Fig. 1:**
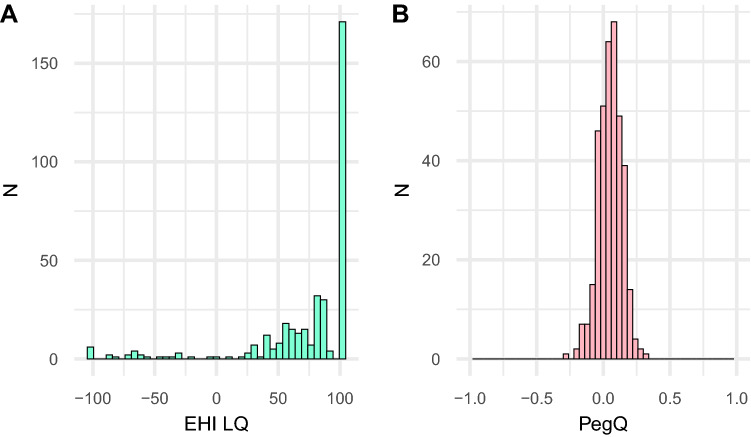
The distribution of handedness. **A** Number of participants (overall *n* = 370) showing a certain laterality quotient (LQ) as measured by EHI. **B** Number of participants (overall *n* = 370) showing a certain PegQ as measured by Pegboard test

Figure 1 shows the distribution of LQs measured via EHI and Pegboard test. The EHI LQ distribution shows a typical J-shape (*M* = 72.48, SD = 43.74), while the PegQ distribution shows a typical bell shape (*M* = 0.06, SD = 0.09). The mean EHI LQ was significantly different from zero and indicated an overall rightward preference (*t*_(369)_ = 31.87, *p* < 0.001). Its range was between − 100 and 100. Overall, there were 26 subjects with an LQ lower than zero (indicating left-handedness) and 343 subjects with an LQ higher than zero (indicating right-handedness). There was one subject with an LQ of 0 (indicating ambidexterity). The mean PegQ indicated a significant rightward preference, as well (*t*_*(*369)_ = 12.34, *p* < 0.001).

### Distribution of EEG alpha asymmetry data

**Fig. 2:**
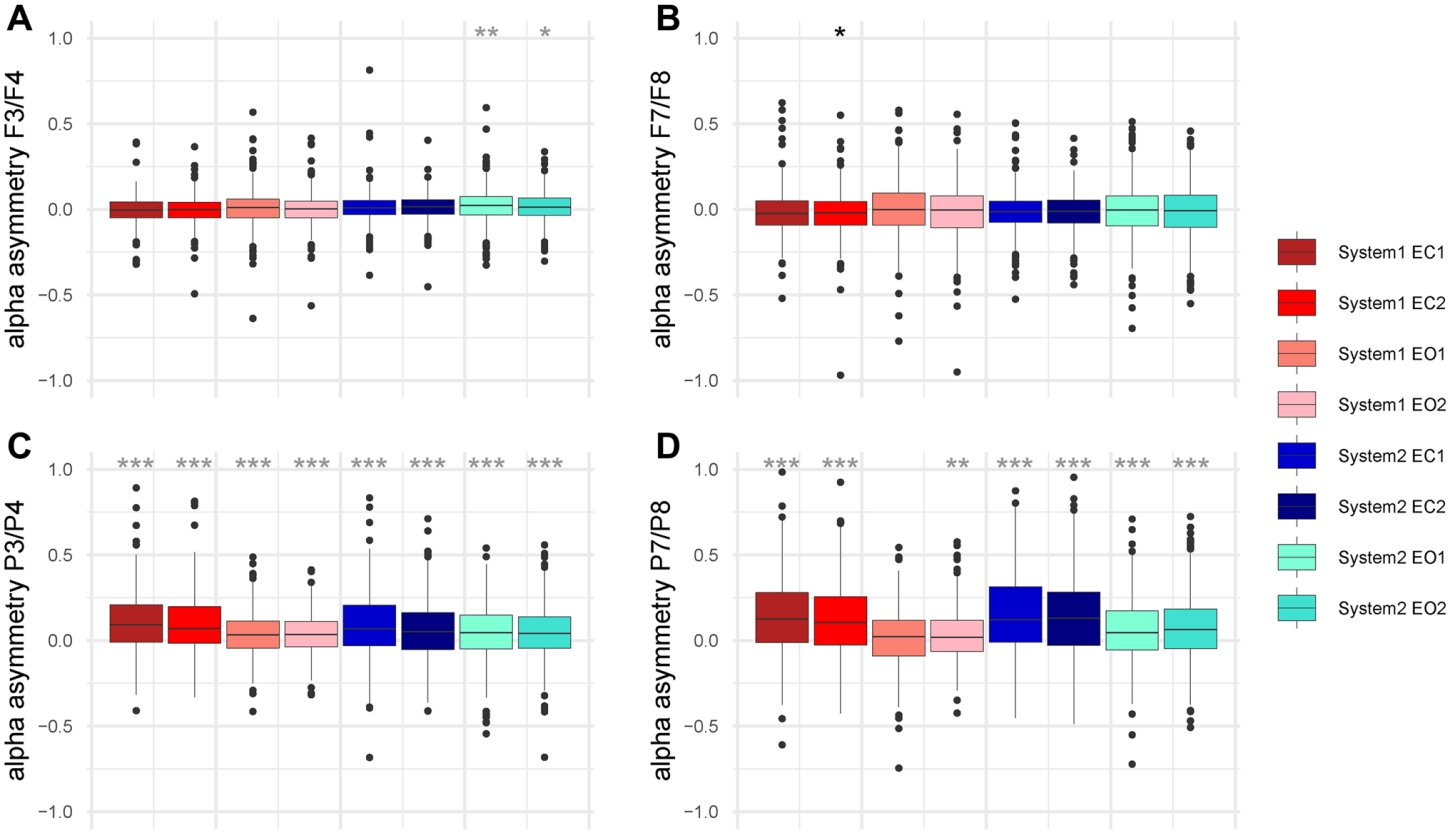
The distribution of alpha asymmetry. The four panels show boxplots of the alpha asymmetry distribution of four electrode pairs over frontal (**A**: F3/F4; **B**: F7/F8) and parietal (**C**: P3/P4; **D**: P7/P8) scalp areas. Boxplots for each of our eight measurements per electrode pair are shown, separately for system 1 (Brain Products) and system 2 (BioSemi), with eyes-open (EO) and eyes-closed (EC), and for session 1 and 2. Dark horizontal lines within the boxplots mark the median. Lower and upper hinges correspond to the 25th and 75th percentile. Whiskers show the 95% confidence intervals. Black dots represent outliers. The asterisks above each boxplot show if this recording’s mean is unequal to zero (*p < 0.05, **p < 0.01, ***p < 0.001). Black asterisks indicate a mean significantly below zero, and gray asterisks indicate a mean significantly above zero

Figure 2 shows the distribution of EEG alpha asymmetry in the eight different recordings per electrode pair (system 1 vs. system 2, session 1 vs. session 2, eyes-open vs. eyes-closed). For a first assessment of the data, we used Bonferroni-corrected *t* -tests against zero to determine whether there was a significant leftward or rightward alpha asymmetry for a specific electrode pair in each condition.

For alpha asymmetry of electrode pair F3/F4, only two measurements’ means were significantly unequal from zero: the session 1 eyes-open recording with system 2 (*t*_(369)_ = 4.06, *p* = 0.002, *M* = 0.022, SD = 0.104) and the session 2 eyes-open recording with system 2 (*t*_(369)_ = 3.51, *p* = 0.017, *M* = 0.016, SD = 0.089). For alpha asymmetry of electrode pair F7/F8, only one measurements’ mean was significantly unequal to zero: the session 2 eyes-closed recording with system 1 (*t*_(369)_ = − 3.31, *p* = 0.034, *M* = − 0.022, SD = 0.127). In contrast, for the electrode pairs P3/P4 and P7/P8, all recordings’ means were unequal from zero, *p* ≤ 0.005, except for P7/P8’s session 1 eyes-open recording with system 1 (*t*_(369)_ = 1.54*, p* = 0.13, *M* = 0.013, SD = 0.17). Thus, while parietal electrode pairs show the expected rightward alpha power asymmetry, frontal electrode pairs did not show the expected leftward alpha power asymmetry, but rather an overall lack of population-level asymmetry. Tables with alpha power in all conditions, the correlation of left and right alpha power and correlations between the two recording systems as well as parietal and frontal electrodes can be found in the supplements (suppl. Table 1–4).

### Reliability of alpha power

Table 1 shows ICC of EEG alpha power for all electrodes and both EEG systems. ICC of alpha power can be considered good to excellent (Cicchetti 1994; Koo and Li 2016). For system 1 (Brain Products), ICC ranges from 0.92 to 0.94 (*M* = 0.93), for in the eyes-closed condition, and it ranges from 0.87 to 0.90 (*M* = 0.89) for the eyes-open condition. ICC for system 2 (BioSemi) ranges from 0.92 to 0.93 (*M* = 0.93) for the eyes-closed condition and 0.9 to 0.93 (*M* = 0.92) for the eyes-open condition. Interestingly, the confidence intervals of system 1 do not overlap between the eyes-closed condition and the eyes-open condition in seven electrodes (all but P4), indicating a higher reliability in the eyes-closed condition. On the other hand, confidence intervals of both conditions overlap in all electrode on system 2. While for the eyes-closed condition confidence intervals of system 1 and system 2 overlap, confidence intervals of three electrodes (F3, F7, and P7) from the eyes-open conditions do not overlap between systems, indicating higher reliability in system 2. Confidence intervals in electrodes on opposite hemispheres always overlap.

**Table 1:** ICC of EEG alpha power for left (F3, F7, P3, P7) and right (F4, F8, P4, P8) electrodes for EEG system 1 (Brain Products) and EEG system 2 (BioSemi) for the eyes-closed (EC) and eyes-open (EO) condition, respectively

	System 1		System 2	
	EC	EO	EC	EO
F3	0.93 (0.92–0.94)	0.89 (0.87–0.90)	0.93 (0.91–0.94)	0.92 (0.91–0.94)
F7	0.93 (0.92–0.94)	0.89 (0.87–0.91)	0.92 (0.91–0.93)	0.93 (0.92–0.94)
P3	0.94 (0.92–0.95)	0.89 (0.87–0.90)	0.93 (0.92–0.94)	0.90 (0.88–0.92)
P7	0.93 (0.92–0.94)	0.87 (0.85–0.89)	0.93 (0.92–0.94)	0.92 (0.91–0.93)
F4	0.93 (0.92–0.94)	0.88 (0.86–0.90)	0.92 (0.91–0.94)	0.91 (0.90–0.93)
F8	0.94 (0.93–0.95)	0.90 (0.88–0.92)	0.92 (0.91–0.93)	0.92 (0.91–0.94)
P4	0.92 (0.91–0.93)	0.90 (0.88–0.91)	0.93 (0.91–0.94)	0.92 (0.90–0.93)
P8	0.93 (0.91–0.94)	0.87 (0.84–0.89)	0.93 (0.91–0.94)	0.90 (0.88–0.91)

The brackets contain the 95% confidence interval of ICC. All ICC show *p* values of *p* < 0.001

### Effect of eye-status and hemisphere on EEG alpha power

To investigate the effect of eye-status on EEG alpha power, we conducted a 2 × 2 × 2 repeated-measure ANOVA. The independent variables were eye-status (eyes-closed, eyes-open), EEG system (system 1, system 2) and hemisphere (left, right), the dependent variable was alpha power. System 1 was the 64-channel EEG system by Brain Products and system 2 was the 32-channel EEG system by BioSemi.

The ANOVA revealed a main effect of hemisphere, *F*_(1,369)_ = 121.01, *p* < 0.001, *η*_p_^2^ = 0.25. Alpha power was higher in the right hemisphere (*M* = 0.73, SD = 0.33) than in the left hemisphere (*M* = 0.69, SD = 0.31). There was also a main effect of system, *F*_(1,369)_ = 182.22, *p* < 0.001, *η*_p_^2^ = 0.33. There was overall higher alpha power in the recording conducted with EEG system 2 (*M* = 0.75, SD = 35) than with EEG system 1 (*M* = 0.67, SD = 0.3). There was also a main effect of eye-status, *F*_(1, 369)_ = 514.62, *p* < 0.001, *η*_p_^2^ = 0.58. Additionally, the interaction between eye-status and hemisphere reached significance, *F*_(1,369)_ = 144.72, *p* < 0.001, *η*_p_^2^ = 0.28. The three-way interaction between eye-status, hemisphere, and system reached significance, as well, *F*_(1, 369)_ = 7.85, *p* = 0.005, *η*_p_^2^ = 0.02. We used Bonferroni-corrected post hoc *t*-tests to investigate the effects further. They revealed that for all systems and eye-status conditions, there was more alpha power in the right (*M* = 0.52–0.94, SD = 0.21–0.47) than in the left hemisphere (*M* = 0.51–0.88, SD = 0.2–0.44), *p* < 0.001. The difference between the hemispheres was larger in the eyes-closed condition (right: *M* = 0.9, SD = 0.45; left: *M* = 0.83, SD = 0.42) than in the eyes-open condition (right: *M* = 0.56, SD = 0.24; left: *M* = 0.55, SD = 0.23), *p* < 0.001. In the eyes-open condition, the difference between right and left hemisphere was smaller in system 1 (right: *M* = 0.52, SD = 0.21; left: *M* = 0.51, SD = 0.20) than system 2 (right: *M* = 0.61, SD = 0.29; left: *M* = 0.59, SD = 0.27), *p* < 0.001.

Taken together, the reliability of alpha power can be considered good to very good. There seems to be more alpha power in the recordings conducted with system 2 than system 1. Alpha power is also higher in the right than the left hemisphere. It is also higher in the eyes-closed condition than in the eyes-open condition.

### Reliability of EEG resting-state alpha asymmetry

Table 2 shows ICC of EEG alpha asymmetry power for all electrode pairs, eye-status conditions, and both EEG systems. ICC of recordings conducted with system 1 (Brain Products) range from 0.68 to 0.87 (*M* = 0.78) in the eye-closed condition and from 0.65 to 0.75 (*M* = 0.71) in the eyes-open condition. ICC of recording conducted with system 2 (BioSemi) range from 0.56 to 0.88 (*M* = 0.76) in the eyes-closed condition and from 0.60 to 0.86 (*M* = 0.73) in the eyes-open condition. Overall, ICC can be considered moderate to good (Cicchetti 1994; Koo and Li 2016). Confidence intervals of the eyes-open condition do not overlap with those of the eyes-closed condition for both systems in parietal electrodes, indicating higher reliability of alpha power asymmetry in the eyes-closed condition for parietal electrodes pairs. Contrary to that, only reliability of frontal electrode pair F7/F8 on system 2 differs between eyes-open and eyes-closed, showing higher reliability in the eyes-open recording. Confidence intervals between systems do not overlap between the F3/F4 eyes-closed (system 1 > system 2) and the F7/F8 eyes-open recordings (system 1 < system 2). Confidence intervals also indicate some difference between electrode pairs. In system 1, frontal electrodes show weaker reliability than parietal electrodes in the eyes-closed condition, and F3/F4 shows weaker reliability than F7/F8 and P7/P8 in the eyes-open condition. Similarly, in system 2, F3/F4 shows weaker reliability than all other electrodes in both conditions. In the eyes-closed condition, P7/P8 shows higher reliability than all other electrode pairs, while in the eyes-open condition, F7/F8 shows higher reliability than all other electrode pairs.

**Table 2:** ICC of EEG alpha asymmetry for all electrode pairs (F3/F4, F7/F8, P3/P4, P7/P8) for EEG system 1 (Brain Products) and EEG system 2 (BioSemi) for the eyes-closed (EC) and eyes-open (EO) condition, respectively

	System 1		System 2	
	EC	EO	EC	EO
F3/F4	0.68 (0.63–0.73)	0.65 (0.60–0.70)	0.56 (0.50–0.62)	0.60 (0.55–0.65)
F7/F8	0.73 (0.69–0.77)	0.76 (0.72–0.79)	0.79 (0.76–0.82)	0.86 (0.83–0.88)
P3/P4	0.83 (0.81–0.86)	0.72 (0.68–0.76)	0.82 (0.79–0.85)	0.72 (0.68–0.76)
P7/P8	0.87 (0.85–0.89)	0.75 (0.71–0.78)	0.88 (0.86–0.90)	0.75 (0.71–0.79)

The brackets contain the 95% confidence interval of ICC. All ICC showed *p* values of *p* < 0.001

Supplementary table 2 shows the correlation between the left and right alpha power in each electrode site and condition. As the correlation between two measures rises, the reliability of the difference score of those measures falls (Clayson et al. 2021).

### Effects of electrode pair and eye-status on alpha asymmetry

To further investigate differences in EEG alpha asymmetry between electrode pairs and different eye-status condition, we conducted one 4 × 2 × 2 repeated-measure ANOVA. The independent variables were electrode pair (F3/F4, F7/F8, P3/P4, P7/P8), EEG system (system 1, system 2), and eye-status (eyes-closed, eyes-open). System 1 is the 64-channel EEG system by Brain Products and system 2 is the 32-channel EEG system by BioSemi.

Mauchly’s test for sphericity showed that the assumption of sphericity had been violated for the main effect of electrode (*W* = 0.5, *p* < 0.001) and all interactions containing the variable electrode pair (*W* = 0.69–0.25, *p* < 0.001). Thus, degrees of freedom and p-values of these effects have been Greenhouse–Geisser corrected.

There was a main effect of electrode pair, *F*_(2.058,759.243)_ = 70.79, *p* < 0.001, *η*_p_^2^ = 0.16. Bonferroni-corrected *t*-tests were calculated to investigate the effects; furthermore, they revealed that all electrode pairs differed from each other, *p* < 0.001. Frontal electrode pair F3/F4 showed a small rightward alpha asymmetry (*M* = 0.008, SD = 0.06), while the F3/F4 pair showed a small leftward alpha asymmetry (*M* = − 0.01, SD = 0.10). Parietal electrode pairs P3/P4 (*M* = 0.06, SD = 0.13) and P7/P8 (*M* = 0.09, SD = 0.16) both showed rightward alpha asymmetry, with P7/P8 showing the stronger one, *p* < 0.001.

There was also a main effect of eye-status, *F*_(1,369)_ = 127.38, *p* < 0.001, *η*_p_^2^ = 0.26. In the eyes-closed condition, there was a stronger overall rightward alpha asymmetry (*M* = 0.05, SD = 0.08) than in the eyes-open condition (*M* = 0.02, SD = 0.07). There was also a main effect of EEG system, *F*_(1,369)_ = 5.16, *p* = 0.024, *η*_p_^2^ = 0.01. Recordings conducted with EEG system 2 show a stronger overall rightward alpha asymmetry (*M* = 0.04, SD = 0.08) than recordings conducted with EEG system 1 (*M* = 0.03, SD = 0.07).

Furthermore, all four interaction effects reached significance, the interaction between electrode pair and eye-status (*F*_(1.723,635.876)_ = 86.99, *p* < 0.001, *η*_p_^2^ = 0.19), the interaction between electrode pair and EEG system (*F*_(2.409,889.011)_ = 6.26, *p* < 0.001, *η*_p_^2^ = 0.02), the interaction between eye-status and EEG system (*F*_(1,369)_ = 18.4, *p* < 0.001, *η*_p_^2^ = 0.05), as well as the three-way interaction between electrode pair, eye-status, and EEG system (*F*_(2.386,880.613)_ = 17.58, *p* < 0.001, *η*_p_^2^ = 0.05). We used Bonferroni-corrected post hoc *t*-test to investigate the effects further. In parietal electrode pairs P3/P4 and P7/P8, there was a larger rightward alpha power asymmetry in the eyes-closed condition (*M* = 0.15–0.07, *SD* = 0.23–0.17) than in the eyes-open condition (*M* = 0.07–0.02, SD = 0.18–0.12), independent of the EEG system used for recording, *p* < 0.001. However, there were more inconsistencies between conditions and systems in the frontal electrode pairs. In electrode pair F3/F4 on EEG system 2, there was a stronger rightward alpha asymmetry in the eyes-open condition (*M* = 0.02, SD = 0.09) than in the eye-closed condition (*M* = 0.005, SD = 0.09), *p* = 0.002. On EEG system 1, however, there was a negative alpha power asymmetry in the eyes-closed condition (*M* = − 0.004, SD = 0.08) and a positive alpha power asymmetry in the eyes-open condition (*M* = 0.005, SD = 0.09), *p* < 0.001, with both means being close to zero. For electrode pair F7/F8 on EEG system 1, there was more leftward alpha power asymmetry in the eyes-closed condition (*M* = − 0.02, SD = 0.12) than in the eyes-open condition (*M* = − 0.003, SD = 0.15), *p* < 0.001. For electrode pair F7/F8 on system 2, there was no evidence for a difference between the eyes-closed (*M* = − 0.01, SD = 0.11) and the eye-open condition (*M* = − 0.004, SD = 0.15), *p* = 0.37.

Taken together, EEG alpha power shows good to excellent reliability, while EEG alpha asymmetry shows moderate to good reliability. Overall, there was a stronger rightward alpha power asymmetry in the recording conducted with EEG system 2 than EEG system 1. Parietal electrodes P3/P4 and P7/P8 show a clear rightward alpha asymmetry, with the one in P7/P8 being stronger. Frontal electrode pair F3/F4 shows positive values, while F7/F8 shows negative values; however, all values are close to zero. The parietal rightward alpha asymmetry is larger in the eyes-closed condition than in the eyes-open condition. There are no consistent effects of condition and EEG on alpha power asymmetry in frontal electrode pairs F3/F4 and F7/F8.

### Association between handedness and EEG resting-state alpha asymmetry

**Fig. 3:**
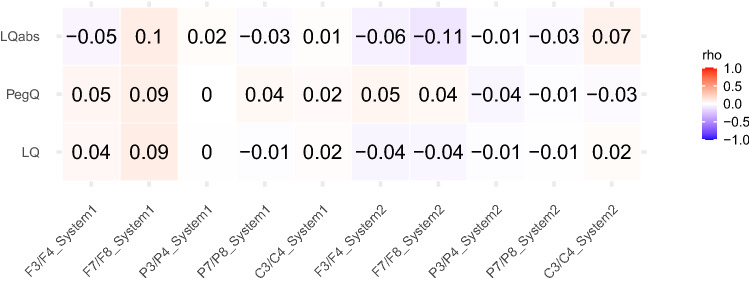
Bayesian correlation heatmap of EEG asymmetry per system and electrode pair (F3/F4, F7/F8, P3/P4, P7/P8, C3/C4), and handedness measures: LQ (EHI), PegQ (Pegboard test), and LQabs (absolute LQ). The calculation was conducted with the mean of all recordings of an electrode pair on the two systems. Positive correlations are shown in red, and negative correlations are shown in blue. The darker the color, the higher the correlation. The number in each square shows the rounded correlation coefficients

Figure 3 shows a heatmap of the median Bayesian correlation between behavioral handedness indexes and alpha power asymmetry averaged across electrode pairs for both EEG systems. In addition to LQ, we also included the absolute LQ (LQabs), hence a measure for strength of handedness without any indication of the direction (example: a LQ of -100 indicating strong left-handedness, would be LQabs of 100, indicating strong handedness). Furthermore, we have added a fifth pair of electrodes to the analysis: C3/C4. These electrodes cover the scalp over sensory–motor areas and have been shown to be linked to handedness. The highest correlation is negative correlation between electrode pair F7/F8 on system 2 and LQabs (*r*_median_ = − 0.11, BF = 1.07), indicating anecdotal evidence in favor of the existence of a correlation. Contrary to that, electrode pair F7/F8 on system 1 shows the highest positive correlation with handedness measures (*r*_median_ = 0.09–0.1, BF = 0.74–0.48) and with that anecdotal evidence in favor of an absence of a correlation. The rest of the correlations (*r*_median_ = − 0.06–0.7, BF = 0.33–0.48) show moderate evidence in favor of an absence of a correlation. An exemplary scatter plot of the correlation between two variables can be found in supplementary Fig. 4.

## Discussion

EEG alpha asymmetry is one of the most widely investigated forms of hemispheric asymmetries (Reznik and Allen 2018). It has been suggested to be a stable trait that has stable long-term associations with motivational and personality traits, but also with psychopathology like MDD (Bruder et al. 1997, 2008; Jesulola et al. 2015; Stewart et al. 2014). In contrast to this widely cited idea, a recent meta-analysis did not find a strong relationship between frontal EEG alpha asymmetry and MDD (van der Vinne et al. 2017). One crucial requirement for replicability of associations between EEG alpha asymmetries and cognitive or clinical variables is a high reliability of the alpha asymmetry LQ. While a body of previous research on EEG alpha asymmetry reliability exists, most studies rely on rather small sample sizes. The aim of the present study was to test the reliability of frontal and parietal EEG alpha asymmetry in a larger dataset, comprising a total of 370 participants with four different EEG recordings on two EEG systems. This allowed us to assess reliability of EEG alpha asymmetry between different sessions within 1 day and recordings with eyes either closed or open. Since all subjects were tested twice with two different EEG systems, we could also investigate if reliability is stable across distinct systems. Furthermore, we aimed to replicate results from previous research concerning overall direction of EEG alpha asymmetry as well as its connection to handedness.

We could replicate the overall rightward alpha asymmetry in parietal electrodes. Surprisingly, we were not able to replicate the overall leftward alpha asymmetry in frontal electrodes. Only one of eight frontolateral (F7/F8) recordings showed significant leftward alpha asymmetry, while two of eight frontomedial (F3/F4) recording showed rightward alpha asymmetry. Findings concerning frontomedial electrodes (F3/F4) are in line with findings by Ocklenburg et al. (2019), who also found no significant alpha asymmetry for electrode pair F3/F4. However, they have reported significant asymmetry for electrode pair F7/F8. This is surprising, as frontomedial (F3/F4) EEG alpha asymmetry has been used as main indicator for frontal asymmetry in a large body of research (Harmon-Jones and Allen 1997; Krzeczkowski et al. 2020; Quaedflieg et al. 2015; Wheeler et al. 1993). Ocklenburg et al. (2019) argued that one of the reasons for the lack of frontomedial (F3/F4) asymmetry may be the specific setup on the EEG cap. This may also be the case in our study. Since we are working with a large cohort study with many experimenters, it can be assumed that there is some variability in the placement of electrodes on the scalp between subjects. Thus, some of the inconsistencies concerning population-level asymmetry might due to inconsistency in electrode positioning. However, in our study, this effect is present in recordings from two different EEG caps, one 64-channel system and one 32-channel system. Thus, it is unlikely that EEG setup is the only reason for the lack of asymmetry on frontal site. One important methodological take-home message from the present study therefore is that researchers should consider including multiple electrode pairs in their analysis when investigating alpha asymmetries.

Another interesting finding of our study concerns the effect of eye movement artifacts on EEG alpha asymmetry. Previous research suggested that the effect of eye movement artifacts on EEG alpha power is minor and that controlling for eye movement artifacts in data pre-processing can be omitted (Hagemann and Naumann 2001). However, there is also a line of research that recommend controlling for eye movement artifacts (Smith et al. 2017). In EEG alpha power asymmetry research, there is no consistency in regard to this matter (Coan and Allen 2003; Meyer et al. 2018; Stewart et al. 2014). The EEG data presented in this manuscript have been analyzed controlling for eye movement artifacts; however, we have conducted the same analysis without controlling for them. The results can be found in the supplements (suppl. Figure 3, suppl. Table 12–13). Distribution of frontal alpha asymmetry differs greatly. While there is clear leftward alpha asymmetry at frontolateral electrodes (F7/F8) when EEG was not controlled for eye movement artifacts (suppl. Figure 3), this leftward alpha asymmetry disappears when controlling for eye movement artifacts (Fig. 1). Parietal alpha asymmetry does not seem to be affected by the choice of analysis. This seems plausible, as frontal electrode sites are much closer to ocular muscles than parietal electrode sites. Furthermore, the analysis without controlling for ocular artifacts produced lower reliability values for the frontomedial (F3/F4) electrode pair, but only in system 2 (see Table 2 and supplementary Table 13). Thus, our data suggest that the choice of controlling or not controlling for eye movement may have a considerable effect on observed alpha power asymmetry, especially in frontal electrode sites. This may be one of the reasons for inconsistent results in the previous research. However, our results have to be interpreted carefully, as we have not investigated the issue systematically. Future research would profit from a study investigating the effect of eye movements on alpha power asymmetry in a large sample.

Our results indicate that eyes-status of the participants—meaning if their eyes were open or closed during the recording—has an effect on alpha power and alpha power asymmetry. Alpha power is generally higher when eyes are closed than when they are open. This effect has been shown in the previous research (Barry et al. 2007) and can be explained by the activity in the visual system which is more active when eyes are open and visual input has to be processed. Additionally, there is more alpha power—or less activity—in the right hemisphere than in the left hemisphere. There is also an effect of eye-status on alpha asymmetry. Furthermore, the direction of this effect is dependent on recording site. Parietal rightward alpha asymmetry was larger in the eyes-closed condition than in the eyes-open condition. In contrast, there is no consistent effect of eye-status on frontal alpha asymmetry, which is unsurprising due to the general lack of asymmetry in this electrode location. These results indicate that the effect of eye-status on alpha asymmetry should be considered when designing an experiment.

We also investigated reliability of absolute EEG alpha power and EEG alpha asymmetry, separately for electrode/electrode pair and eye-status condition. For EEG alpha power, the ICC for the Brain Products system ranged between 0.92 and 0.94 in the eyes-closed condition and from 0.87 to 0.90 in the eyes-open condition, and ICC for the BioSemi system ranged from 0.92 to 0.93 in the eyes-closed condition and from 0.9 to 0.93 in the eyes-open condition. Thus, alpha power reliability can be rated as good to excellent, depending on interpretation criteria (Cicchetti 1994; Koo and Li 2016). Reliability does not differ between the two different hemispheres. Reliability is higher in the eyes-closed condition, but only in system 1.

For EEG alpha asymmetry, ICC for the Brain Products system ranged from 0.86 to 0.87 in the eyes-closed and from 0.65 to 0.75 in the eyes-open condition. ICC for the BioSemi system ranged from 0.56 to 0.88 in the eyes-closed condition and from 0.60 to 0.86 in the eyes-open condition. It can be rated as moderate to good, depending on interpretation criteria (Cicchetti 1994; Koo and Li 2016). Similar to alpha power, there was higher reliability in the eyes-closed condition than in the eyes-open condition, but only in parietal electrode pairs. The frontolateral (F7/F8) electrode pair showed higher reliability in the eyes-open condition, however only in the BioSemi system. Thus, while there seems to be a rather constant effect of eye-status on reliability of parietal alpha asymmetry, this effect is not consistent for frontal alpha asymmetry and may even be reversed for frontolateral electrodes. The frontomedial (F3/F4) recording site showed the weakest reliability across both systems and conditions, thereby replicating the results reported by Koller-Schlaud et al. (2020). Thus, alpha asymmetry in frontomedial electrodes does seem to yield comparably low reliability in both resting-state and task EEG. However, we could not replicate the weak reliability of the parietolateral (P7/P8) electrode pair. These results further harden our suggestions to not rely on frontomedial EEG alpha asymmetry alone but to include other electrodes as well.

We have also investigated if reliability of alpha power and alpha power asymmetry differ between age groups (suppl. Tables 5–10). Our results show no clear pattern of differences between age groups and that alpha power and alpha power reliability show a good reliability in young adults, middle-aged adults, and older adults. These results are in line with the previous research reporting stable alpha power asymmetry in the elderly (Mathewson et al. 2015).

The choice of EEG system also has an effect on alpha power and alpha asymmetry. Generally, there was higher alpha power in the BioSemi system than in the Brain Products system. There was also a stronger rightward alpha asymmetry in the BioSemi system than in the Brain Products system. However, it is hard to draw any conclusion from that finding. Since we are comparing a 32-channel system to a 64-channel system, there are differences in the placement of the electrodes. Furthermore, the two amplifiers differ in the internal reference systems they use (see “Methods”). It cannot be excluded that differences in alpha power are (at least in part) due to these different set-ups. However, differences in reliability were considerable small and reliability overall good in both systems. Thus, we do not recommend any of the systems over the other.

As far as we know, our study presents the largest EEG alpha asymmetry reliability study so far. However, our study does not come without limitations. The first thing to consider is that reliability in our study cannot automatically be transferred to other studies, due to a large number of differences in method and samples between studies. Thus, we recommend that all authors investigating alpha power asymmetry should calculate reliability in their own sample. If their study design does not allow calculations of test–retest reliability, they could use internal consistency as a measure of reliability instead. Studies of investigation internal consistency of alpha power asymmetry have reported Cronbach’s alpha approaching 0.90 (Allen et al. 2004; Hill et al. 2020; Towers and Allen 2009). Towers and Allen (2009) present a detailed discussion of estimating internal consistency in EEG asymmetry scores. Additionally, our results are hard to apply to an experiment conducting an emotional task instead of resting state. According to the capability model (Coan et al. 2006), EEG alpha asymmetry during an emotion-inducing task is a much better predictor of motivational or psychopathological variables associated with frontal activity. It appears plausible that EEG alpha asymmetry during a specific task may be more reliable than in sheer resting-state. Another important thing to consider is that we only tested healthy participants, but that EEG alpha asymmetries are an index that are also often used in clinical populations. Even though a previous study has found no difference in alpha asymmetry reliability between healthy and depressed populations (Vuga et al. 2006), our results may not apply for clinical cohorts. Furthermore, our analysis concerns short-term reliability of recordings conducted on the same day and our results may not be applicable to long-term reliability of EEG alpha power and EEG alpha asymmetry.

We could not replicate the link between strong right-handedness and alpha asymmetry as presented by Ocklenburg et al. (2019). There was only one electrode pair that showed a small link to handedness degree, namely the frontolateral (F7/F8) electrode pair on system 2. Surprisingly, the negative correlation indicates that stronger handedness (without considering the direction of this handedness) is associated with stronger leftward alpha asymmetry, or stronger rightward frontal activity. However, all other electrode sites yielded results indicative of no link between handedness degree, handedness direction, hand performance, and alpha asymmetry. Thus, our overall results indicate no relationship between alpha asymmetry and handedness. Interestingly, additional electrode pair C3/C4 also showed no link to alpha asymmetry in contrast to previous findings (Papousek and Schulter 1999). One reason for those inconsistencies may be the small number of left-handed participants in the present study. Only 26 (7%) of our 370 participants had an LQ indicative of left-handedness, which is a very low amount in comparison to 64 or 25% of 235 subjects in Ocklenburg et al. (2019) and 26 or 51% of 51 subjects in Packheiser et al. (2020). While handedness is considered one of the most prominent phenotypes of neuroanatomic lateralization, only 10–30% of left-handers show altered functional hemispheric organization (Westerhausen et al. 2006). As described by van der Haegen et al. (2013), not handedness as such, but altered functional hemispheric organization in left-handers produces differences between left and right-handers. Apart from having a small sample of left-handers to begin with, we also have no way of knowing how many of the left-handers show altered functional hemispheric organization. Thus, it is not surprising that our study did not yield any effect in contrast to studies that have deliberately oversampled left-handed individuals.

## Conclusion

This study presents the largest frontal and parietal EEG alpha asymmetry reliability study so far. We found good reliability of EEG alpha power and EEG alpha asymmetry on both investigated EEG systems. Reliability of alpha asymmetry in frontomedial electrode pairs was weaker than in other electrode sites. Frontal EEG alpha asymmetry seems to be less stable than parietal EEG alpha asymmetry. Furthermore, the eye-statuses of participants, meaning if their eyes are closed or opened during the recording, have a considerable effect on EEG alpha power and EEG alpha asymmetry and may lower the reliability if not controlled for in the study design. The general lack and low reliability of frontal EEG alpha asymmetry may present a reason for inconsistent results in alpha asymmetry research. Further studies investigating the reliability in clinical cohorts as well as during emotion-inducing tasks are needed to evaluate and interpret the results of experimental studies.

## Supplementary Information

Below is the link to the electronic supplementary material.

Supplementary file1 (DOCX 559 KB)

## Declarations

### Availability of data and materials

The data that support the findings of this study was not acquired by the corresponding author, but is part of the Dortmund Vital Study and was provided by IfADo. Data are however available from the authors upon reasonable request and with permission of IfADo.

### Code availability

Available on request.
