# Supplementary material for: Frontal and parietal EEG alpha asymmetry: a large-scale investigation of short-term reliability on distinct EEG systems
Source: Brain Struct Funct. 2021 Oct 21;227(2):725–40. doi: 10.1007/s00429-021-02399-1 (PMC8843903; doi:10.1007/s00429-021-02399-1)
Supplement: Supplementary file 1 — Supplementary file1 (DOCX 559 KB) [file 429_2021_2399_MOESM1_ESM.docx]

**Supplementary Material**

1. **Alpha power in all conditions**

**Suppl. Table 1** Alpha power of all left sided (F3, F7, P3, P7) and right sided electrodes (F4, F8, P4, P8) for all conditions (eyes closed vs. eyes open, session 1 vs. session 2) and for EEG system 1 (Brain Products) and EEG system 2 (BioSemi).

|  | system 1 | | | | system 2 | | | | |
| --- | --- | --- | --- | --- | --- | --- | --- | --- | --- |
|  | eyes closed | | eyes open | | eyes closed | | eyes open | | |
|  | session 1 | session 2 | session 1 | session 2 | session 1 | session 2 | session 1 | session 2 |  |
| F3 | 0.68 | 0.72 | 0.43 | 0.49 | 0.79 | 0.82 | 0.52 | 0.57 |  |
| F7 | 0.67 | 0.71 | 0.46 | 0.52 | 0.79 | 0.82 | 0.57 | 0.62 |  |
| P3 | 0.78 | 0.84 | 0.46 | 0.51 | 0.83 | 0.88 | 0.53 | 0.59 |  |
| P7 | 0.93 | 1.01 | 0.55 | 0.63 | 1.00 | 1.08 | 0.62 | 0.69 |  |
| F4 | 0.68 | 0.72 | 0.44 | 0.49 | 0.79 | 0.83 | 0.53 | 0.58 |  |
| F8 | 0.65 | 0.69 | 0.46 | 0.51 | 0.77 | 0.81 | 0.56 | 0.60 |  |
| P4 | 0.88 | 0.93 | 0.47 | 0.54 | 0.90 | 0.93 | 0.56 | 0.62 |  |
| P8 | 1.12 | 1.17 | 0.57 | 0.66 | 1.19 | 1.27 | 0.68 | 0.77 |  |

1. **Correlation of left and right alpha power**

**Suppl. Table 2** Spearman’s rank correlation of alpha power in the left and right hemisphere in for both systems and for both eye-status conditions.

|  | system 1 | | system 2 | |
| --- | --- | --- | --- | --- |
|  | eyes-closed | eyes-open | eyes-closed | eyes-open |
| F3/F4 | 0.99*** | 0.96*** | 0.99*** | 0.98*** |
| F7/F8 | 0.97*** | 0.91*** | 0.97*** | 0.93*** |
| P3/P4 | 0.96*** | 0.96*** | 0.95*** | 0.95*** |
| P7/P8 | 0.93*** | 0.94*** | 0.92*** | 0.93*** |

*** p < .001

1. **Correlation between the recording systems**

**Suppl. Table 3** Spearman’s rank correlation of alpha power and alpha power asymmetry for the eyes-closed (EC) and eyes-open (EO) recordings between both systems. All correlations reached a p-value of p < .001.

|  | alpha power | | | | | | | | alpha power asymmetry | | | |
| --- | --- | --- | --- | --- | --- | --- | --- | --- | --- | --- | --- | --- |
|  | F3 | F7 | P3 | P7 | F4 | F8 | P4 | P8 | F3/F4 | F7/F8 | P3/P4 | P8/P7 |
| EC | 0.94 | 0.92 | 0.92 | 0.93 | 0.94 | 0.93 | 0.92 | 0.91 | 0.46 | 0.42 | 0.63 | 0.58 |
| EO | 0.91 | 0.86 | 0.9 | 0.9 | 0.9 | 0.87 | 0.9 | 0.9 | 0.35 | 0.36 | 0.47 | 0.45 |

1. **Correlation of alpha power asymmetry between frontal and parietal electrodes**

**Suppl. Table 4** Spearman’s rank correlation of alpha power asymmetry between frontal and parietal electrodes in the eyes-closed (EC) and eyes-open (EO) condition on both systems.

|  | system 1 | | | | system 2 | | | |
| --- | --- | --- | --- | --- | --- | --- | --- | --- |
|  | P3/P4 | | P7/P8 | | P3/P4 | | P7/P8 | |
|  | EC | EO | EC | EO | EC | EO | EC | EO |
| F3/F4 | -0.26*** | 0.11* | -0.26*** | -0.06 | -0.34*** | -0.18*** | -0.28*** | -0.02 |
| F7/F8 | -0.23*** | -0.11* | -0.18*** | -0.03 | -0.19*** | -0.12* | -0.06 | 0.09 |

* p < .05, *** p < .001

1. **Frequency spectra of electrodes**

**Suppl. Figure 1** Frequency spectra of frontal (F3, F7, F4, F8) and parietal (P3, P7, P4, P8) electrodes for the eyes-closed (A, B) and eyes-open (C, D) condition on both EEG systems.

1. **Alpha power asymmetry reliability divided by age groups**

There is a large body of research showing that aging has a considerable effect on lateralization of brain activity (Hirnstein et al., 2013; Ocklenburg & Güntürkün, 2018). Alpha power and alpha asymmetry have been shown to be affected by aging as well, with older adults showing reduced hemispheric asymmetry than younger adults (Deiber et al., 2013; Hong et al., 2015; Huizeling et al., 2021; Kolev et al., 2002; Vaden et al., 2012). Interestingly, studies have shown that while alpha power asymmetry in the elderly are stable (Mathewson et al., 2015), the association of alpha power and psychopathological measures could not be replicated in an older sample (Carvalho et al., 2011). Since our study comprises subjects of a very wide age-span (20 -70 years), effects of age on the reliability reported cannot be ruled out. Thus, we have conducted the following analysis to investigate the influence of age on reliability of alpha asymmetry. For this we have divided our sample into three groups: young adults (age 20-35, M = 27.52, SD = 4.09, n = 120), middle-aged adults (age 36-55, M = 47.25, SD = 5.71, n = 163) and older adults (age 56 – 70, M = 62.6, SD = 4.48, n = 87)

- 1. **Reliability of alpha power divided by age groups**

Supplementary tables 5-7 show the reliability of alpha power divided by age group. The confidence intervals of ICC for the electrodes all overlap except in four cases (P4, system 1, EC: young > middle; P8, system 2, EO: young > middle & old > middle; P8, system 1, EC: old > middle). However, discrepancies are very small (0.01 – 0.04) and there is no clear pattern that reliability of one condition, electrode or system differs between the age groups.

**Suppl. Table 5** ICC of EEG alpha power in young adults (20 - 35) for left (F3, F7, P3, P7) and right (F4, F8, P4, P8) electrodes for EEG system 1 (Brain Products) and EEG system 2 (BioSemi) and for the eyes-closed (EC) and eyes-open (EO) condition. Brackets show the 95% confidence interval. P-values for all ICC are p < 0.001.

**Young adults**

|  | system 1 | | | system 2 | | | | |
| --- | --- | --- | --- | --- | --- | --- | --- | --- |
|  | EC | EO | | | EC | | EO | |
| F3 | 0.93 (0.90 - 0.95) | | 0.89 (0.86 - 0.92) | | | 0.93 (0.90 - 0.94) | | 0.93 (0.90 - 0.94) |
| F7 | 0.94 (0.92 - 0.96) | | 0.91 (0.88 - 0.93) | | | 0.93 (0.91 - 0.95) | | 0.94 (0.92 - 0.95) |
| P3 | 0.93 (0.91 - 0.95) | | 0.88 (0.84 - 0.91) | | | 0.93 (0.91 - 0.95) | | 0.92 (0.89 - 0.94) |
| P7 | 0.92 (0.89 - 0.94) | | 0.86 (0.81 - 0.89) | | | 0.92 (0.89 - 0.94) | | 0.92 (0.89 - 0.94) |
| F4 | 0.93 (0.90 - 0.95) | | 0.90 (0.87 - 0.93) | | | 0.92 (0.89 - 0.94) | | 0.91 (0.88 - 0.93) |
| F8 | 0.94 (0.92 - 0.95) | | 0.90 (0.87 - 0.93) | | | 0.92 (0.90 - 0.94) | | 0.93 (0.90 - 0.95) |
| P4 | 0.94 (0.92 - 0.96) | | 0.90 (0.86 - 0.92) | | | 0.94 (0.92 - 0.95) | | 0.92 (0.89 - 0.94) |
| P8 | 0.92 (0.90 - 0.94) | | 0.85 (0.80 - 0.88) | | | 0.93 (0.91 - 0.95) | | 0.92 (0.89 - 0.94) |

**Suppl. Table 6** ICC of EEG alpha power in middle-aged adults (36 - 55) for left (F3, F7, P3, P7) and right (F4, F8, P4, P8) electrodes for EEG system 1 (Brain Products) and EEG system 2 (BioSemi) and for the eyes-closed (EC) and eyes-open (EO) condition. Brackets show the 95% confidence interval. P-values for all ICC are p < 0.001.

**Middle-aged adults**

|  | system 1 | | | system 2 | | | | |
| --- | --- | --- | --- | --- | --- | --- | --- | --- |
|  | EC | EO | | | EC | | EO | |
| F3 | 0.93 (0.90 - 0.94) | | 0.86 (0.83 - 0.89) | | | 0.91 (0.88 - 0.93) | | 0.91 (0.88 - 0.91) |
| F7 | 0.92 (0.90 - 0.94) | | 0.87 (0.83 - 0.90) | | | 0.90 (0.88 - 0.92) | | 0.92 (0.89 - 0.93) |
| P3 | 0.94 (0.92 - 0.95) | | 0.88 (0.85 - 0.91) | | | 0.92 (0.90 - 0.94) | | 0.88 (0.85 - 0.91) |
| P7 | 0.93 (0.91 - 0.95) | | 0.87 (0.83 - 0.90) | | | 0.92 (0.90 - 0.94) | | 0.90 (0.88 - 0.92) |
| F4 | 0.93 (0.91 - 0.94) | | 0.85 (0.81 - 0.89) | | | 0.91 (0.89 - 0.92) | | 0.90 (0.88 - 0.93) |
| F8 | 0.94 (0.92 - 0.95) | | 0.90 (0.87 - 0.92) | | | 0.91 (0.88 - 0.93) | | 0.91 (0.89 - 0.93) |
| P4 | 0.89 (0.86 - 0.91) | | 0.89 (0.86 - 0.91) | | | 0.89 (0.86 - 0.92) | | 0.89 (0.86 - 0.91) |
| P8 | 0.90 (0.88 - 0.92) | | 0.85 (0.81 - 0.88) | | | 0.90 (0.88 - 0.92) | | 0.85 (0.80 - 0.88) |

**Suppl. Table 7** ICC of EEG alpha power in older adults (56 - 70) for left (F3, F7, P3, P7) and right (F4, F8, P4, P8) electrodes for EEG system 1 (Brain Products) and EEG system 2 (BioSemi) and for the eyes-closed (EC) and eyes-open (EO) condition. Brackets show the 95% confidence interval. P-values for all ICC are p < 0.001.

**Older adults**

|  | system 1 | | | system 2 | | | | |
| --- | --- | --- | --- | --- | --- | --- | --- | --- |
|  | EC | EO | | | EC | | EO | |
| F3 | 0.93 (0.90 - 0.95) | | 0.89 (0.85 - 0.92) | | | 0.94 (0.91 - 0.96) | | 0.93 (0.90 - 0.95) |
| F7 | 0.92 (0.89 - 0.95) | | 0.90 (0.85 - 0.93) | | | 0.93 (0.90 - 0.95) | | 0.94 (0.92 - 0.96) |
| P3 | 0.91 (0.87 - 0.93) | | 0.88 (0.84 - 0.92) | | | 0.92 (0.89 - 0.94) | | 0.90 (0.86 - 0.93) |
| P7 | 0.96 (0.94 - 0.97) | | 0.89 (0.85 - 0.93) | | | 0.93 (0.90 - 0.95) | | 0.95 (0.92 - 0.96) |
| F4 | 0.92 (0.89 - 0.94) | | 0.88 (0.82 - 0.92) | | | 0.93 (0.90 - 0.95) | | 0.88 (0.83 - 0.91) |
| F8 | 0.93 (0.90 - 0.95) | | 0.90 (0.85 - 0.93) | | | 0.93 (0.90 - 0.95) | | 0.93 (0.90 - 0.95) |
| P4 | 0.91 (0.88 - 0.94) | | 0.88 (0.83 - 0.92) | | | 0.93 (0.90 - 0.95) | | 0.93 (0.90 - 0.95) |
| P8 | 0.95 (0.93 - 0.96) | | 0.90 (0.86 - 0.93) | | | 0.93 (0.90 - 0.95) | | 0.94 (0.92 - 0.96) |

- 1. **Reliability of alpha power asymmetry divided by age groups**

Supplementary tables 8-10 show the reliability of alpha power asymmetry divided by age groups. There are several recordings were confidence intervals of ICC do not overlap between the age groups. For electrode pair F3/F4 young adults and middle-aged adults show higher reliability than older adults, however only in the eyes-closed condition on system 2. This is due to the older adults showing a comparatively low reliability (ICC = 0.40) in this recording. In electrode pair F7/F8 on system 1 older adults show higher reliability than middle-aged adults in both conditions and higher than young adults in the eye-closed condition. In electrode pair P3/P4 on system 2 in the eyes-closed condition older adults show lower reliability than middle-aged and young adults. Thus, differences are not consistent across systems, conditions or electrodes and show no clear pattern.

**Suppl. Table 8** ICC of EEG alpha asymmetry in young adults (20 - 35) for all electrode pairs (F3/F4, F7/F8, P3/P4, P7/P8) for EEG system 1 (Brain Products) and EEG system 2 (BioSemi) for the eyes-closed (EC) and eyes-open (EO) condition, respectively. The brackets contain the 95% confidence interval of ICC. All ICC showed p-values of p < .001.

**Young adults**

|  | system 1 | | system 2 | |
| --- | --- | --- | --- | --- |
|  | EC | EO | EC | EO |
| F3/F4 | 0.71 (0.62 - 0.78) | 0.66 (0.57 - 0.74) | 0.69 (0.60 - 0.76) | 0.62 (0.52 - 0.71) |
| F7/F8 | 0.70 (0.61 - 0.77) | 0.75 (0.68 - 0.81) | 0.76 (0.69 - 0.82) | 0.84 (0.78 - 0.88) |
| P3/P4 | 0.87 (0.83 - 0.90) | 0.77 (0.70 - 0.83) | 0.87 (0.82 - 0.90) | 0.82 (0.76 - 0.86) |
| P7/P8 | 0.88 (0.84 - 0.91) | 0.73 (0.65 - 0.79) | 0.90 (0.87 - 0.93) | 0.78 (0.72 - 0.83) |

**Suppl. Table 9** ICC of EEG alpha asymmetry in middle-aged adults (36 - 55) for all electrode pairs (F3/F4, F7/F8, P3/P4, P7/P8) for EEG system 1 (Brain Products) and EEG system 2 (BioSemi) for the eyes-closed (EC) and eyes-open (EO) condition, respectively. The brackets contain the 95% confidence interval of ICC. All ICC showed p-values of p < .001.

**Middle-aged adults**

|  | system 1 | | system 2 | |
| --- | --- | --- | --- | --- |
|  | EC | EO | EC | EO |
| F3/F4 | 0.67 (0.59 - 0.74) | 0.63 (0.55 - 0.70) | 0.66 (0.59 - 0.73) | 0.63 (0.54 - 0.70) |
| F7/F8 | 0.70 (0.62 - 0.76) | 0.71 (0.64 - 0.77) | 0.78 (0.72 - 0.82) | 0.86 (0.82 - 0.89) |
| P3/P4 | 0.82 (0.77 - 0.85) | 0.73 (0.66 - 0.78) | 0.83 (0.78 - 0.87) | 0.66 (0.58 - 0.73) |
| P7/P8 | 0.85 (0.81 - 0.88) | 0.74 (0.67 - 0.79) | 0.88 (0.85 - 0.91) | 0.70 (0.63 - 0.76) |

**Suppl. Table 10** ICC of EEG alpha asymmetry in older adults (56 - 70) for all electrode pairs (F3/F4, F7/F8, P3/P4, P7/P8) for EEG system 1 (Brain Products) and EEG system 2 (BioSemi) for the eyes-closed (EC) and eyes-open (EO) condition, respectively. The brackets contain the 95% confidence interval of ICC. All ICC showed p-values of p < .001.

**Older adults**

|  | system 1 | | system 2 | |
| --- | --- | --- | --- | --- |
|  | EC | EO | EC | EO |
| F3/F4 | 0.68 (0.57 - 0.76) | 0.70 (0.60 - 0.78) | 0.40 (0.24 - 0.53) | 0.54 (0.40 - 0.65) |
| F7/F8 | 0.84 (0.78 - 0.88) | 0.86 (0.81 - 0.90) | 0.85 (0.79 - 0.89) | 0.87 (0.82 - 0.91) |
| P3/P4 | 0.81 (0.74 - 0.86) | 0.63 (0.51 - 0.73) | 0.67 (0.56 - 0.76) | 0.69 (0.58 - 0.77) |
| P7/P8 | 0.89 (0.85 - 0.92) | 0.77 (0.69 - 0.83) | 0.84 (0.78 - 0.89) | 0.78 (0.70 - 0.84) |

- 1. **Influence of age on alpha power and alpha power asymmetry**

To further investigate the effect of age on alpha power and alpha power asymmetry, we have conducted the ANOVAs from the manuscript with the addition of age as a further factor.

*Alpha power*

To investigate the effect of age on alpha power we conducted a 2x2x2x3 repeated measure ANOVA. The independent within-variables were eye-status (eyes-closed vs. eyes-open), EEG system (system 1 vs. system 2) and hemisphere (left vs. right), the independent between-variable was age group (young adults vs. middle-aged adults vs. older adults).

Since significances and effect sizes of the effects reported in the manuscript (3.4. Effect of eye-status and hemisphere on EEG alpha power) did not change considerably by adding age as variable, only effects with the variable age are reported here. There was a main effect of age group, *F*_(1,367)_ = 17,31, *p* < .001, η_p_^2^ = 0.09. Bonferroni-corrected post-hoc t-test revealed that all age groups differed from one another, p < .001. Young adults showed strongest alpha power (*M* = 0.84, *SD* = 0.35), followed by middle-aged adults (*M* = 0.67, *SD* = 0.30) and older adults (*M* = 0.61, *SD* = 0.25). There was also a significant interaction between age group and hemisphere, *F*_(1,367)_ = 8,87, *p* < .001, η_p_^2^ = 0.05. Bonferroni-corrected post-hoc t-tests revealed that in all age groups there was a significant difference between alpha power on the left and alpha power on the right side, p < .001, with alpha power always being more prominent in the right hemisphere. The difference between hemispheres in young adults (left: M = 0.81, SD = 0.34, right: M = 0.87, SD = 0.36) was larger than in middle-aged adults (left: M = 0.65, SD = 0.29, right: M = 0.69, SD = 0.31), p = .004, and older adults (left: M = 0.60, SD = 0.25, right: M = 0.62, SD = 0.26), p < .001.

There was also a significant interaction between age group and eye-status, F(1,367) = 11,59, p < .001, η_p_2 = 0.06. Bonferroni-corrected post-hoc t-test revealed that the eye-closed and eye-open condition differed from each other in all age groups, p ≤ .013. The size of the difference declined with age, with young adults (open: M = 0.65, SD = 0.26, closed: M = 1.04, SD = 0.48) showing larger differences than middle-aged adults (open: M = 0.52, SD = 0.22, closed: M = 0.82, SD = 0.39), p = .016, and older adults (open: M = 0.50, SD = 0.19, closed: M = 0.72, SD = 0.33) p < .001, and middle-aged adults also showing larger differences than older adults, p = .034.

There was also a significant interaction between age group and system, F(1,367) = 13,43, p < .001, η_p_2 = 0.07. Bonferroni-corrected post-hoc t-test revealed that alpha power differed between the two systems in all age-groups, p < .001. The difference between the two systems was larger in younger adults (system 1: M = 0.78, SD = 0.32, system 2: M = 0.91, SD = 0.39) than in middle-aged adults (system 1: M = 0.63, SD = 0.28, system 2: M = 0.28, SD = 0.32), p < .001, and older adults (system 1: M = 0.59, SD = 0.25, system 2: M = 0.63, SD = 0.27), p < 001.

*Alpha power asymmetry*

To further investigate the effect of age on alpha asymmetry, we conducted one 4x2x2x3 repeated measure ANOVA. The independent within variables were electrode pair (F3/F4 vs. F7/F8 vs. P3/P4 vs. P7/P8), EEG system (system 1 vs. system 2) and eye-status (eyes-closed vs. eyes-open), the independent between variable was age group (young adults vs. middle-aged adults vs. older adults).

Since significances and effect sizes of the effects reported in the manuscript (3.6. Effects of electrode pair, EEG system and eye-status on alpha asymmetry) did not change considerably by adding age as variable, only effects with the variable age are reported here. There was a main effect of age group, *F*_(1,367)_ = 5.58, *p* = .004, η_p_^2^ = 0.03. Bonferroni-corrected t-tests revealed that young adults (*M* = 0.06, *SD* = 0.07) showed stronger rightward alpha asymmetry than middle-aged adults (*M* = 0.03, *SD* = 0.07) and older adults (*M* = 0.03, *SD* = 0.06), p > 001.

There was also a significant interaction between age group and electrode pair *F*_(4.11,754,42)_ = 5.58, *p* < .001, η_p_^2^ = 0.03. Bonferroni-corrected post-hoc t-test were conducted to investigate the interaction. In electrode pair F3/F4 older adults show stronger rightward alpha power asymmetry (*M* = 0.02, *SD* = 0.06) than younger adults (*M* = 0.007, *SD* = 0.06) and middle-aged adults (*M* = 0.002, *SD* = 0.07). In electrode pair F7/F8, no evidence for differences between age groups was present, p = 0.45 - 1. In electrode pair P3/P4 young adults showed a stronger rightward alpha asymmetry (*M* = 0.08, *SD* = 0.14) than middle-aged adults (*M* = 0.05, *SD* = 0.13), p < .001. In electrode pair P7/P8 all age groups differed from each other, p < .001, with younger adults showing the strongest rightward alpha asymmetry (*M* = 0.14, *SD* = 0.17), followed by middle-aged adults (*M* = 0.08, *SD* = 0.15) and older adults (*M* = 0.05, *SD* = 0.14).

Taken together, there seems to be little effect of age on the reliability of alpha power and rather inconsistent effects of age on the reliability of alpha power asymmetry. The ANOVAs showed, that there is an overall decline of alpha power and alpha power asymmetry with age in some electrode sites, but not all. However, one has to keep in mind that effect sizes were rather small, even though they reached significance.

1. **Reliability of other electrode sites**

Even though our manuscript focuses on alpha power asymmetry in frontal and parietal sites, the data set included further electrode sites. Here we present the distribution of alpha power asymmetry and the reliability of alpha power asymmetry in seven further electrode sites: Fp1/Fp2, FC3/FC4, CP3/CP4, T7/T8, C3/C4, PO3/P4, O1/O2. These electrode pairs were chosen since they were available for both the 64-channel and the 32-channel EEG system.

**Suppl. Figure 2** The Distribution of alpha asymmetry**.** The four panels show boxplots of the alpha asymmetry distribution of seven electrode pairs (A: Fp1/Fp2, B: FC3/FC4, C: CP3/CP4, D: T7/T8, E: C3/C4, F: PO3/PO4, G: O1/O2) over different scalp areas. Boxplots for each of our eight measurements per electrode pair are shown, separately for system 1 (Brain Products) and system 2 (BioSemi), with eyes open (EO) and eyes closed (EC), and for session 1 and 2. Dark horizontal lines within the boxplots mark the median. Lower and upper hinges correspond to the 25^th^ and 75^th^ percentile. Whiskers show the 95% confidence intervals. Black dots represent outliers. The asterisks above each boxplot show if this recording’s mean is unequal to zero (* p<.05, ** p<.01, *** p<.001). Black asterisks indicate a mean significantly below zero, and gray asterisks indicate a mean significantly above zero

**Suppl. Table 11:** ICC of EEG alpha asymmetry for further electrode pairs (Fp1/Fp2, FC3/FC4, CP3/CP4, T7/T8, C3/C4, PO3/PO4, O1/O2) for EEG system 1 (Brain Products) and EEG system 2 (BioSemi) and eyes-closed (EC) and eyes-open (EO), respectively. Brackets show the 95% confidence interval. All ICC have p-values of p < .001.

|  | system 1 | | | | system 2 | | |
| --- | --- | --- | --- | --- | --- | --- | --- |
|  | EC | | EO | | EC | | EO |
| Fp1/Fp2 | 0.69 (0.64 - 0.73) | 0.81 (0.78 - 0.84) | | 0.76 (0.72 - 0.79) | | 0.82 (0.79 - 0.84) | |
| FC3/FC4 | 0.50 (0.43 - 0.56) | 0.49 (0.42 - 0.55) | | 0.69 (0.64 - 0.73) | | 0.61 (0.55 - 0.66) | |
| CP3/CP4 | 0.75 (0.71 - 0.79) | 0.65 (0.59 - 0.69) | | 0.76 (0.72 - 0.80) | | 0.72 (0.67 - 0.76) | |
| T7/T8 | 0.77 (0.73 - 0.80) | 0.76 (0.73 - 0.80) | | 0.73 (0.69 - 0.77) | | 0.73 (0.69 - 0.77) | |
| C3/C4 | 0.72 (0.68 - 0.76) | 0.57 (0.51 - 0.63) | | 0.69 (0.64 - 0.73) | | 0.63 (0.58 - 0.68) | |
| PO3/PO4 | 0.72 (0.68 - 0.76) | 0.49 (0.42 - 0.55) | | 0.80 (0.77 - 0.83) | | 0.74 (0.70 - 0.77) | |
| O1/O2 | 0.82 (0.79 - 0.84) | 0.27 (0.19 - 0.35) | | 0.77 (0.73 - 0.80) | | 0.68 (0.64 - 0.73) | |

1. **Results without controlling for ocular artifacts in the data analysis**

In addition to the analysis reported in the manuscript, we have also conducted the same analysis, but without controlling for eye movement artifacts in the EEG data analysis. Past research has suggested, that alpha power and alpha power asymmetry are relatively unaffected by ocular movements and that controlling for those artifacts in data processing is optional (Hagemann & Naumann, 2001). However, other papers suggest otherwise (Smith et al., 2017). While the following analysis shows that reliability of alpha power and alpha power asymmetry, which is the focus of our manuscript, is relatively unaffected by controlling for ocular artifact, there are some considerable differences in frontal alpha asymmetry. The implications and limitations of this are discussed in the manuscript.

- 1. **Distribution of EEG alpha asymmetry data**

Supplementary figure 3 shows the distribution of EEG alpha asymmetry in the eight different recordings per electrode pair (system 1 vs. system 2, session 1 vs. session 2, eyes-open vs. eyes-closed). For a first assessment of the data, we used Bonferroni-corrected t-tests against zero to determine whether there was a significant leftward or rightward alpha asymmetry for a specific electrode pair in each condition.

For alpha asymmetry of electrode pair F3/F4 only one measurement’s mean was significantly unequal from zero (*t*_(387)_ = -3.31, *p* = .034), namely the session 2 eyes-open recording with system 1 (*M* = -0.015, *SD* = 0.088). In contrast, for the electrode pairs F7/F8, P3/P4 and P7/P8 all recordings’ means were unequal from zero, p ≤ .007, except for P7/P8’s session 1 eyes-open recording with system 1 (*t*_(387)_ = 1.6*, p* = 1, *M* = 0.013, *SD* = 0.16). Furthermore, frontal electrodes on average showed a leftward alpha power asymmetry (*M* = -0.021; range -0.338 to 0.139, *SD* = 0.06), while parietal electrodes showed a rightward alpha power asymmetry (*M* = 0.078; range -0.247 to 0.575, *SD* = 0.127).

**Suppl. Fig. 3** The Distribution of alpha asymmetry**.** The four panels show boxplots of the alpha asymmetry distribution of four electrode pairs over frontal (A: F3/F4; B: F7/F8) and parietal (C: P3/P4; D: P7/P8) scalp areas. Boxplots for each of our eight measurements per electrode pair are shown, separately for system 1 (Brain Products) and system 2 (BioSemi), with eyes open (EO) and eyes closed (EC), and for session 1 and 2. Dark horizontal lines within the boxplots mark the median. Lower and upper hinges correspond to the 25^th^ and 75^th^ percentile. Whiskers show the 95% confidence intervals. Black dots represent outliers. The asterisks above each boxplot show if this recording’s mean is unequal to zero (* p<.05, ** p<.01, *** p<.001). Black asterisks indicate a mean significantly below zero, and gray asterisks indicate a mean significantly above zero

- 1. **Reliability of alpha power**

Supplementary table 12 shows ICC of EEG alpha power for all electrodes and both EEG systems. Depending on classification criteria, ICC of alpha power can be considered good to excellent (Cicchetti, 1994; Koo & Li, 2016). For system 1 (Brain Products) ICC from 0.92 to 0.94 in the eyes-closed condition (*M* = 0.93), while it ranges from 0.87 to 0.90 (M = 0.89) in the eyes-open condition. ICC for system 2 (BioSemi) is similar, ranging from 0.92 to 0.93 (M = 0.93) in the eyes-closed condition and 0.90 to 0.93 (M = 0.92) in the eyes-open condition.

**Suppl. Table 12:** ICC of EEG alpha power for left (F3, F7, P3, P7) and right (F4, F8, P4, P8) electrodes for EEG system 1 (Brain Products) and EEG system 2 (BioSemi) for the eyes-closed (EC) and eyes-open (EO) condition, respectively. The brackets contain the 95% confidence interval of ICC. All ICC show p-values of p < 0.001.

|  | System 1 | | | System 2 | | | | |
| --- | --- | --- | --- | --- | --- | --- | --- | --- |
|  | EC | EO | | | EC | | EO | |
| F3 | 0.93 (0.92 - 0.94) | | 0.89 (0.87 - 0.91) | | | 0.93 (0.91 - 0.94) | | 0.93 (0.92 - 0.94) |
| F7 | 0.93 (0.92 - 0.94) | | 0.89 (0.87 - 0.91) | | | 0.92 (0.91 - 0.93) | | 0.93 (0.91 - 0.94) |
| P3 | 0.94 (0.93 - 0.95) | | 0.90 (0.88 - 0.91) | | | 0.93 (0.92 - 0.94) | | 0.90 (0.89 - 0.92) |
| P7 | 0.94 (0.92 - 0.94) | | 0.88 (0.86 - 0.90) | | | 0.93 (0.92 - 0.94) | | 0.93 (0.92 - 0.94) |
| F4 | 0.93 (0.92 - 0.94) | | 0.89 (0.87 - 0.91) | | | 0.92 (0.91 - 0.94) | | 0.92 (0.91 - 0.93) |
| F8 | 0.94 (0.93 - 0.95) | | 0.90 (0.88 - 0.92) | | | 0.92 (0.91 - 0.93) | | 0.93 (0.91 - 0.94) |
| P4 | 0.92 (0.91 - 0.93) | | 0.90 (0.89 - 0.92) | | | 0.93 (0.92 - 0.94) | | 0.92 (0.91 - 0.94) |
| P8 | 0.92 (0.91 - 0.94) | | 0.87 (0.85 - 0.89) | | | 0.93 (0.91 - 0.94) | | 0.91 (0.89 - 0.92) |

- 1. **Effect of eye-status and hemisphere on EEG alpha power**

To investigate the effect of eye-status on EEG alpha power we conducted a 2x2x2 repeated measure ANOVA. The independent variables were eye-status (eyes-closed vs. eyes-open), EEG system (system 1 vs. system 2) and hemisphere (left vs. right), the dependent variable was alpha power. System 1 is the 64-channel EEG system by Brain Products and system 2 is the 32-channel EEG system by BioSemi.

The ANOVA revealed a main effect of hemisphere, *F*_(1,387)_ = 95.36, *p* < .001, η_p_^2^ = 0.20. Alpha power was higher in the right hemisphere (*M* = 0.75, *SD* = 0.33) than in the left hemisphere (*M* = 0.72, *SD* = 0.31). There was also a main effect of system, *F*_(1,387)_ = 146.23, *p* < .001, η_p_^2^ = 0.27. There overall higher alpha power in the recordings conducted with EEG system 2 (*M* = 0.77, *SD* = 0.35) than with EEG system 1 (*M* = 0.7, *SD* = 0.3). There was also a main effect of eye-status, *F*_(1,387)_ = 495.67, *p* < .001, η_p_^2^ = 0.56. Alpha power was higher in the eyes-closed condition (*M* = 0.88, *SD* = 0.42) than in the eyes-open condition (*M* = 0.59, *SD* = 0.23).

Additionally, the interaction between eye-status and hemisphere reached significance, *F*_(1,387)_ = 161.58, *p* < .001, η_p_^2^ = 0.29, as well as the interaction between system and hemisphere, *F*_(1,387)_ = 10.56, *p* = .001, η_p_^2^ = 0.03. The three-way interaction between eye-status, hemisphere and system reached significance as well, *F*_(1,387)_ = 13.28, *p* < .001, η_p_^2^ = 0.03. The difference between the hemispheres was larger in the eyes-closed condition (right: *M* = 0.91, *SD* = 0.44; left: *M* = 0.85, *SD* = 0.41) than in the eyes-open condition (right: *M* = 0.59, *SD* = 0.24; left: *M* = 0.58, *SD* = 0.23), p < .001. The difference between hemispheres was larger in system 2 (right: *M* = 0.91, *SD* = 0.44; left: *M* = 0.85, *SD* = 0.41) than system 1, p < .001. In the eyes-open condition, the difference between right and left hemisphere was larger in system 2 (right: *M* = 0.79, *SD* = 0.361; left: *M* = 0.75, *SD* = 0.34) than system 1 (right: *M* = 0.71, *SD* = 0.31; left: *M* = 0.68, *SD* = 0.28), p < .001. However, there was no evidence for a difference between left and right hemisphere in the eyes-open recordings on system 1 (right: *M* = 0.55, *SD* = 0.21; left: *M* = 0.55, *SD* = 0.20), p = 0.39.

Taken together the reliability of alpha power can be considered good to very good. There seems to be more alpha power in the recordings conducted with system 2 than system 1. Alpha power is also higher in the right than the left hemisphere. It is also higher in the eyes-closed condition than in the eyes-open condition.

- 1. **Reliability of EEG resting state alpha asymmetry**

Supplementary table 13 shows ICC of EEG alpha asymmetry power for all electrode pairs and both EEG systems. ICC of recordings conducted with system 1 (Brain Products) range from 0.68 to 0.87 (*M* = 0.76) in the eyes-closed condition and from 0.70 to 0.75 (*M* = 0.73) in the eyes-open condition. ICC of recording conducted with system 2 (BioSemi) range from 0.56 to 0.89 (*M* = 0.72) in the eyes-closed condition and from 0.61 to 0.75 (*M* = 0.70) in the eyes-open condition. Overall, ICC can be considered average to good (Cicchetti, 1994; Koo & Li, 2016).

**Suppl. Table 13:** ICC of EEG alpha asymmetry for all electrode pairs (F3/F4, F7/F8, P3/P4, P7/P8) for EEG system 1 (Brain Products) and EEG system 2 (BioSemi) for the eyes-closed (EC) and eyes-open (EO) condition, respectively. The brackets contain the 95% confidence interval of ICC. All ICC showed p-values of p < .001.

|  | System 1 | | System 2 | |
| --- | --- | --- | --- | --- |
|  | EC | EO | EC | EO |
| F3/F4 | 0.68 (0.63 - 0.72) | 0.70 (0.65 - 0.74) | 0.56 (0.50 - 0.62) | 0.61 (0.55 - 0.66) |
| F7/F8 | 0.71 (0.67 - 0.75) | 0.74 (0.70 - 0.77) | 0.63 (0.58 - 0.68) | 0.73 (0.69 - 0.77) |
| P3/P4 | 0.84 (0.81 - 0.86) | 0.72 (0.68 - 0.76) | 0.81 (0.78 - 0.84) | 0.72 (0.67 - 0.75) |
| P7/P8 | 0.87 (0.85 - 0.89) | 0.75 (0.71 - 0.78) | 0.89 (0.87 - 0.91) | 0.75 (0.72 - 0.79) |

- 1. **Effects of electrode pair and eye-status on alpha asymmetry**

To further investigate differences in EEG alpha asymmetry between electrode pairs and different eye-status condition, we conducted one 4x2x2 repeated measure ANOVA. The independent variables were electrode pair (F3/F4, F7/F8, P3/P4, P7/P8), EEG system (system 1 vs. system 2) and eye-status (eyes-closed, eyes-open). System 1 is the 64-channel EEG system by Brain Products and system 2 is the 32-channel EEG system by BioSemi.

Mauchly’s test for sphericity showed that the assumption of sphericity had been violated for the main effect of electrode (W = 0.38, p < .001) and all interactions containing the variable electrode pair (W = 0.69 – 0.23, p < .001). Thus, degrees of freedom and p-values of these effects have been Greenhouse-Geisser corrected.

There was a main effect of electrode pair, *F*_(1.932,747.684)_ = 127.35, *p* < .001, η_p_^2^ = 0.25. Bonferroni-corrected t-tests revealed that all electrode pairs differed from each other, p < .001. Frontal electrode pair F3/F4’s mean is negative and close to zero (*M* = -0.0007, *SD* = 0.06), while the F7/F8 pair shows larger leftward alpha asymmetry (*M* = -0.04, *SD* = 0.08), p < .001. Parietal electrode pairs P3/P4 (*M* = 0.06, *SD* = 0.13) and P7/P8 (*M* = 0.09, *SD* = 0.16) both showed rightward alpha asymmetry, with P7/P8 showing the stronger one, p < .001.

There was also a main effect of eye-status, *F*_(1,387)_ = 168.81, *p* < .001, η_p_^2^ = 0.30. In the eyes-closed condition there was a stronger overall rightward alpha asymmetry (*M* = 0.05, *SD* = 0.08) than in the eyes-open condition (*M* = 0.01, *SD* = 0.06). There was also a main effect of EEG system, *F*_(1,387)_ = 24.52, *p* < .001, η_p_^2^ = 0.06. Recordings conducted with EEG system 2 shows a stronger overall rightward alpha asymmetry (*M* = 0.04, *SD* = 0.08) than EEG system 1 (*M* = 0.02, *SD* = 0.07).

Furthermore all four interaction effects reached significance, the interaction between electrode pair and eye-status (*F*_(1.734,671.058)_ = 67.13, *p* < .001, η_p_^2^ = 0.15), the interaction between electrode pair and EEG system (*F*_(2.265,876.555)_ = 10.07, *p* < .001, η_p_^2^ = 0.03), the interaction between eye-status and EEG system (*F*_(1,387)_ = 39.69, *p* < .001, η_p_^2^ = 0.09), as well as the three-way interaction between electrode pair, eye-status and EEG system (*F*_(2.462,952.705)_ = 4.18, *p* < .001, η_p_^2^ = 0.01). We used Bonferroni-corrected post-hoc t-test to investigate the effects further. In frontal electrode F7/F8 there was a larger leftward alpha asymmetry in the eyes-open condition (*M* = -0.06 – - 0.02, *SD* = 0.12 – 0.11) than in the eyes-closed condition (*M* = -0.04 – -0.02, *SD* = 0.09 – 0.08), independent of the EEG system, p ≤ .042. In frontal electrode F3/F4 there was no evidence for a difference between the eyes-closed (*M* = -0.006 – 0.01, *SD* = 0.07) and the eyes-open condition (*M* = -0.01 – 0.01, *SD* = 0.09 – 0.08), p = 1. In parietal electrode pairs P3/P4 and P7/P98 there was a larger rightward alpha power asymmetry in the eyes-closed condition (*M* = 0.15 – 0.07, *SD* = 0.24 – 0.17) than in the eyes-open condition (*M* = 0.07 – 0.02, *SD* = 0.17 – 0.11), independent of the EEG system used for recording, p < .001. The difference was larger in system 1 (closed: *M* = 0.1 – 0.13, *SD* = 0.21 – 0.17, open: *M* = 0.04 – 0.02, *SD* = 0.14 – 0.11) than in system 2 (closed: *M* = 0.15 – 0.07, *SD* = 0.24 – 0.18, open: *M* = 0.07 – 0.04, *SD* = 0.17 – 0.16), p < .001.

Taken together, EEG alpha power shows good to very good reliability while EEG alpha asymmetry shows average to good reliability. There is a frontal leftward alpha asymmetry and a parietal rightward asymmetry. These tendencies, however, seem to be weaker in the electrode pairs F3/F4 and P3/P4 than F7/F8 and P7/P8. The parietal rightward alpha asymmetry is larger in the eyes-closed condition than in the eyes-open condition. The frontal leftward alpha asymmetry is stronger in the open-eye condition in electrode pair F7/F8, however there is no evidence for a difference between eyes-closed and eyes-open condition in electrode pair F3/F4.

1. **Exemplary scatter plots**

Supplementary figure 4 shows an exemplary scatterplot of the correlation between the first F3/F4 eyes-open recording on system 1 and the second F3/F3 eyes-open recording on system 1. Supplementary figure 6B shows a scatterplot of the correlation between F3/F4 recordings from system 1 and the LQ (depicted in figure 3).


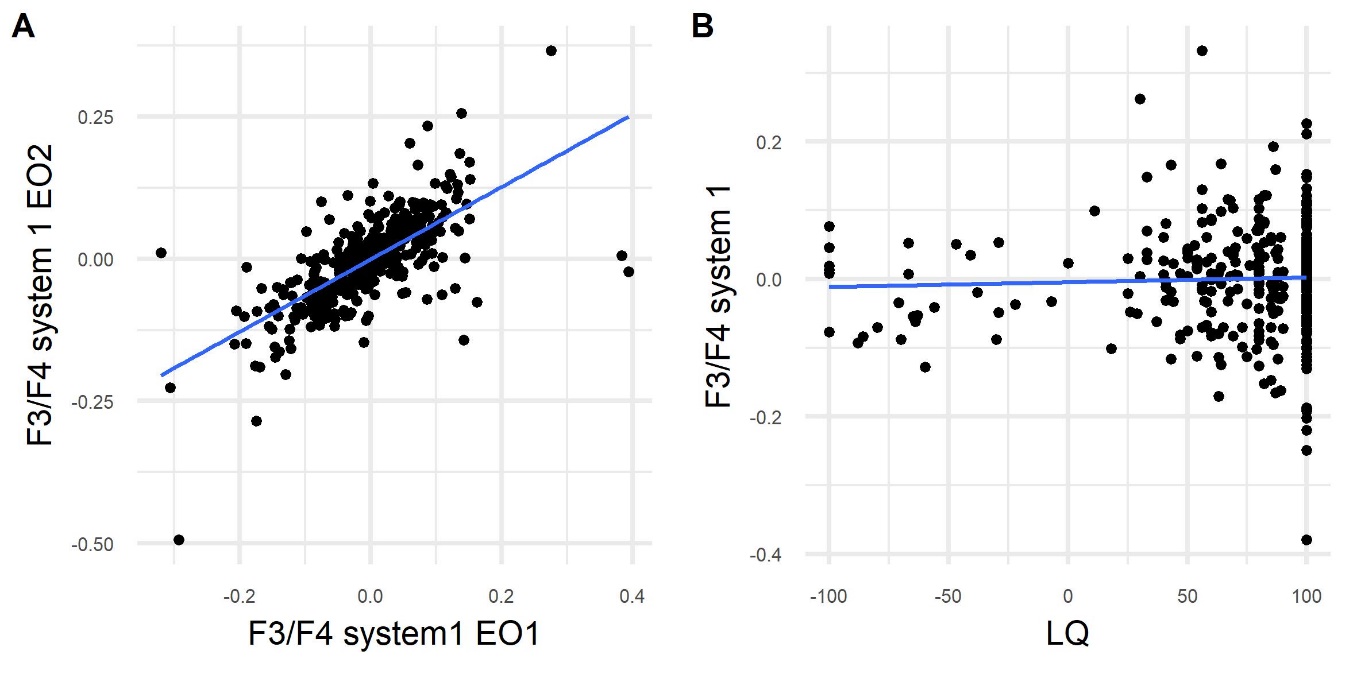


**Suppl. Figure 4** Exemplary scatter plots of correlations between two alpha asymmetry measurements (F3/F4 system 1 session 1 eyes-open and F3/F4 system 1 session 2 eyes-open) and figure 3 (association of alpha power asymmetry and handedness). The blue regression line is computed with the two variables shown in the scatter plot, respectively.

References

Carvalho, A., Moraes, H., Silveira, H., Ribeiro, P., Piedade, R. A. M., Deslandes, A. C., Laks, J., & Versiani, M. (2011). Eeg frontal asymmetry in the depressed and remitted elderly: Is it related to the trait or to the state of depression? *Journal of Affective Disorders*, *129*(1-3), 143–148. https://doi.org/10.1016/j.jad.2010.08.023

Cicchetti, D. V. (1994). Guidelines, criteria, and rules of thumb for evaluating normed and standardized assessment instruments in psychology. *Psychological Assessment*, *6*(4), 284–290. https://doi.org/10.1037/1040-3590.6.4.284

Deiber, M.-P., Ibañez, V., Missonnier, P., Rodriguez, C., & Giannakopoulos, P. (2013). Age-associated modulations of cerebral oscillatory patterns related to attention control. *NeuroImage*, *82*, 531–546. https://doi.org/10.1016/j.neuroimage.2013.06.037

Hagemann, D., & Naumann, E. (2001). The effects of ocular artifacts on (lateralized) broadband power in the eeg. *Clinical Neurophysiology*, *112*(2), 215–231. https://doi.org/10.1016/S1388-2457(00)00541-1

Hirnstein, M., Westerhausen, R., Korsnes, M. S., & Hugdahl, K. (2013). Sex differences in language asymmetry are age-dependent and small: A large-scale, consonant-vowel dichotic listening study with behavioral and fmri data. *Cortex; a Journal Devoted to the Study of the Nervous System and Behavior*, *49*(7), 1910–1921. https://doi.org/10.1016/j.cortex.2012.08.002

Hong, X., Sun, J., Bengson, J. J., Mangun, G. R., & Tong, S. (2015). Normal aging selectively diminishes alpha lateralization in visual spatial attention. *NeuroImage*, *106*, 353–363. https://doi.org/10.1016/j.neuroimage.2014.11.019

Huizeling, E., Wang, H., Holland, C., & Kessler, K. (2021). Changes in theta and alpha oscillatory signatures of attentional control in older and middle age. *The European Journal of Neuroscience.* Advance online publication. https://doi.org/10.1111/ejn.15259

Kolev, V., Yordanova, J., Basar-Eroglu, C., & Basar, E. (2002). Age effect on visual eeg responses reveal distinct frontal alpha networks, *113*, 901–910. https://doi.org/10.1016/S1388-2457(02)00106-2

Koo, T. K., & Li, M. Y. (2016). A guideline of selecting and reporting intraclass correlation coefficients for reliability research. *Journal of Chiropractic Medicine*, *15*(2), 155–163. https://doi.org/10.1016/j.jcm.2016.02.012

Mathewson, K. J., Hashemi, A., Sheng, B., Sekuler, A. B., Bennett, P. J., & Schmidt, L. A. (2015). Regional electroencephalogram (eeg) alpha power and asymmetry in older adults: A study of short-term test-retest reliability. *Frontiers in Aging Neuroscience*, *7*, 177. https://doi.org/10.3389/fnagi.2015.00177

Ocklenburg, S., & Güntürkün, O. (2018). *The lateralized brain: The neuroscience and evolution of hemispheric asymmetries*. Academic Press.

Smith, E. E., Reznik, S. J., Stewart, J. L., & Allen, J. J. B. (2017). Assessing and conceptualizing frontal eeg asymmetry: An updated primer on recording, processing, analyzing, and interpreting frontal alpha asymmetry. *International Journal of Psychophysiology : Official Journal of the International Organization of Psychophysiology*, *111*, 98–114. https://doi.org/10.1016/j.ijpsycho.2016.11.005

Vaden, R. J., Hutcheson, N. L., McCollum, L. A., Kentros, J., & Visscher, K. M. (2012). Older adults, unlike younger adults, do not modulate alpha power to suppress irrelevant information. *NeuroImage*, *63*(3), 1127–1133. https://doi.org/10.1016/j.neuroimage.2012.07.050
